# Obligately Tungsten-Dependent Enzymes—Catalytic Mechanisms, Models and Applications

**DOI:** 10.1021/acs.biochem.5c00116

**Published:** 2025-05-05

**Authors:** Maciej Szaleniec, Johann Heider

**Affiliations:** † Jerzy Haber Institute of Catalysis and Surface Chemistry, Polish Academy of Sciences, 30-239 Krakow, Poland; ‡ Faculty of Biology, 9377 Philipps-Universität Marburg, 35037 Marburg, Germany; § Center for Synthetic Microbiology, Philipps-Universität Marburg, 35037 Marburg, Germany

**Keywords:** tungsten enzymes, aldehyde oxidoreductase, formaldehyde oxidoreductase, glyceraldehyde-3-phosphate ferredoxin oxidoreductases, acetylene hydratase, benzoyl-CoA reductase, AOR, WOR, FOR, GOR, GAPOR, BamB, metallopterin

## Abstract

Tungsten-dependent enzymes incorporate a tungsten ion into their active site in the form of a complex with two pyranometallopterin (MPT) molecules, also known as tungsten cofactor (W-co). W-co-containing enzymes are found in several bacteria and archaea, predominantly in enzymes involved in anaerobic metabolism. While some enzymes occur with either molybdenum or tungsten in their active sites, we concentrate here on enzymes obligately depending on W-co, which are not functional as isoenzymes with Mo-co. These are represented by several subtypes of aldehyde oxidoreductases (AORs), class II benzoyl-CoA reductase (BCRs) and acetylene hydratase (AHs). They catalyze either low-potential redox reactions or the unusual hydration reaction of acetylene. In this review, we analyze the catalytic and structural properties of these enzymes and focus on various mechanistic hypotheses proposed to describe their catalytic action, including hypothetical mechanistic patterns common to all of these enzymes. The biochemical characterization of the enzymes is supported by studies with functional inorganic models that help in the elucidation of their spectroscopic and catalytic features. Finally, we discuss a range of ongoing biotechnological applications utilizing obligately tungsten-dependent enzymes in producing value-added chemicals, indicating the expected advantages of incorporating these enzymes into biotechnological processes despite their intrinsic oxygen-sensitivity and the requirement of special recombinant expression platforms.

## Graphical abstract

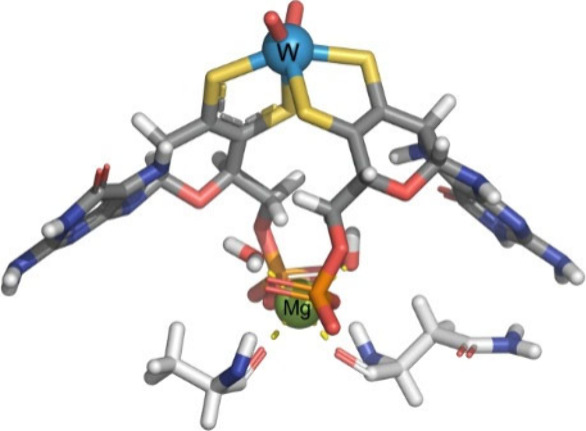

## Introduction

Tungsten is the heaviest element known to play an essential role in biochemistry. It occurs in the so-called tungstoenzymes exclusively in ligation to two three-ring pyranopterin cofactors. Because the same cofactors are also used in molybdoenzymes, we are using the recently introduced alternative term ‘metallopterins’^1^ throughout this review, together with the terms Mo-co or W-co for the entire molybdenum or tungsten cofactors. While the metallopterin-dependent enzymes are affiliated to four unrelated families, only two of these actually contain known tungstoenzymes: the DMSO reductase (DMSOR) family consists mostly of molybdoenzymes, but also contains some tungstoenzymes, whereas the aldehyde (tungsten) oxidoreductase family consists almost exclusively of tungstoenzymes (recently reviewed in^1–4^ ). The latter family has previously been called either AOR or WOR family,^1,5^ although not exclusively consisting of AORs or tungsten-containing enzymes, therefore we will refer to it as AOR/WOR family. Despite the differences in sequence and composition, all tungstoenzymes share a similar active site layout, consisting of a tungsten-*bis*-metallopterin cofactor joined by at least one close-by Fe_4_S_4_ cluster, as schematically illustrated in Figure 1. Most tungstoenzymes affiliated with the DMSOR family share high similarities to molybdoenzymes of the same functionality. The respective Mo- or W-dependent versions occur either as orthologs in different bacterial or archaeal species or as paralogues in the same organism, which are usually differentially produced in response to Mo or W availability. This is particularly the case for many formate dehydrogenases and formyl-methanofuran dehydrogenases, but also for some additional molybdoenzymes.^1,6^ The only obligately W-dependent enzyme in the DMSOR family is acetylene hydratase (AH), which is also the only known Mo- or W-dependent enzyme not catalyzing a redox reaction. The other obligately W-dependent enzymes are affiliated to the AOR/WOR family, namely class II benzoyl-CoA reductases involved in anaerobic degradation of aromatic compounds (BCR), different types of aldehyde oxidoreductases (AORs *sensu lato*) and a recently discovered AOR with an additional function as acylsulfonate hydroxylase (WOR5_*Pf*_/ASOR). The principal properties of these enzymes are summarized in Table S1. The big advantage of using W rather than Mo apparently comes into play for redox reactions at very low potentials. Because of the filled-in 4d and 4f electron shells in W compared to Mo, the electrons of the inner shells experience a relativistic mass increase combined with orbital contraction, explaining why Mo and W are highly similar in their atomic radii and other chemical parameters, although being in different periods of the periodic table. In the case of the valence electrons, the relativistic effects differ for the s and p orbitals, compared to the d and f orbitals of W: the former experience orbital contraction, and the latter orbital expansion because of their larger distance to the nucleus. These effects are believed to cause the generally lower observed redox potentials of W species, compared to the analogous Mo redox couples.^7^ Because there are already reviews available on the general properties of facultatively W-containing enzymes, the differences between Mo-and W-containing isoenzymes^1,8,9^ and the biosynthesis of Mo- or W-cofactors,^10,11^ we concentrate here on the obligately W-dependent enzymes, comparing their underlying reaction mechanisms. In addition, we add some recent material on chemical models for W-biocatalysis and present potential biotechnical applications.

**Figure 1:**
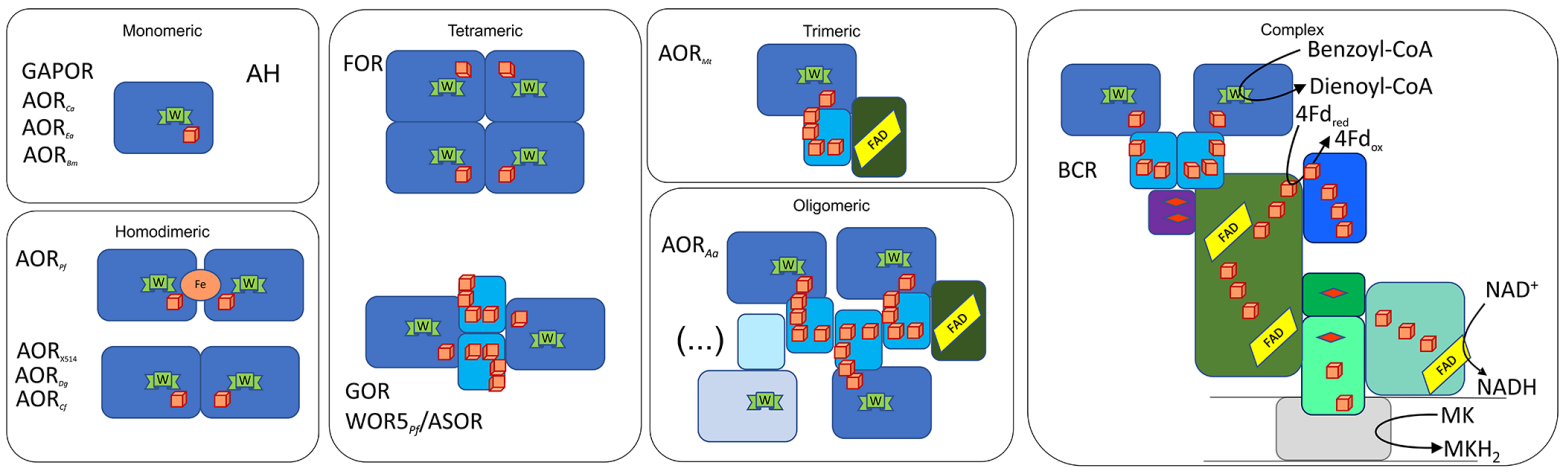
Schematic representation of subunit organization in obligately tungsten-dependent enzymes. W-co is depicted as green ribbon, Fe_4_S_4_ or Fe_2_S_2_ clusters as orange cubes or diamonds, and flavins as yellow rhomboids; AOR – aldehyde oxidoreductase, AH – acetylene hydratase, WOR5_Pf_/ASOR – aliphatic sulfonate ferredoxin oxidoreductase, BCR – class II benzoyl-CoA reductases, FOR – formaldehyde oxidoreductase, GAPOR from archaea or GOR from bacteria – glyceraldehyde-3-phosphate oxidoreductases.

## AOR/WOR Family

The AOR/WOR family (N-terminal domains represented by cl08354 in the conserved protein domain database) consists of enzymes from many strictly or facultatively anaerobic *Bacteria* or *Archaea* that share marked similarity between themselves but differ fundamentally from the members of the other superfamilies of Mo- or W-dependent enzymes.^1^ A phylogenetic tree was constructed from 395 full sequences of the catalytic subunits of a representative collection of AOR/WOR family members (see SI). All known members contain a conserved W- or Mo-*bis*-metallopterin cofactor in their active centers, with a bridging Mg^2+^ ion involved in binding the phosphate groups of both metallopterins and anchoring the cofactor in the enzyme. The family is divided into several subclades according to sequence conservation, structure of the enzyme complexes, and catalyzed reactions (Figure 1, 2). While many of the clades still represent purely hypothetical enzymes (Figure 2), several of the clades can be assigned to functions based on the biochemical or structural characterization of at least one member or allow some reasonable predictions about their functionality from their operon structures. Only one subclade, represented by enzymes encoded in the genomes of many strictly or facultatively anaerobic bacteria (e.g., YdhV from E. coli), appears to prefer a redox-active Mo-*bis*-MPT cofactor. While it is still unclear what biochemical reaction YdhV performs, it contains Cys336 as an additional ligand to the metal ion but lacks the second shell amino acids usually conserved in AORs.^12^ A closely related clade represents the catalytic subunit of class II benzoyl-CoA reductases (BCR), which shares the same additional Cys ligand and the lack of otherwise conserved second shell residues with the YdhV clade, while all other AOR/WOR clades with characterized members represent variations of aldehyde-oxidizing redox enzymes without a protein-based ligand at their W-cofactors. The best-known representatives of the latter clades are aldehyde oxidoreductases with broad substrate spectra (AOR *sensu stricto*), formaldehyde oxidoreductases (FOR), glyceraldehyde-3-phosphate oxidoreductases (GAPOR from *Archaea* or GOR from *Bacteria*) and additional broad-spectrum aldehyde oxidoreductases provisionally named W-dependent oxidoreductase (WOR4_*Pf*_, WOR5_*Pf*_, and others; see Figure 2).

**Figure 2:**
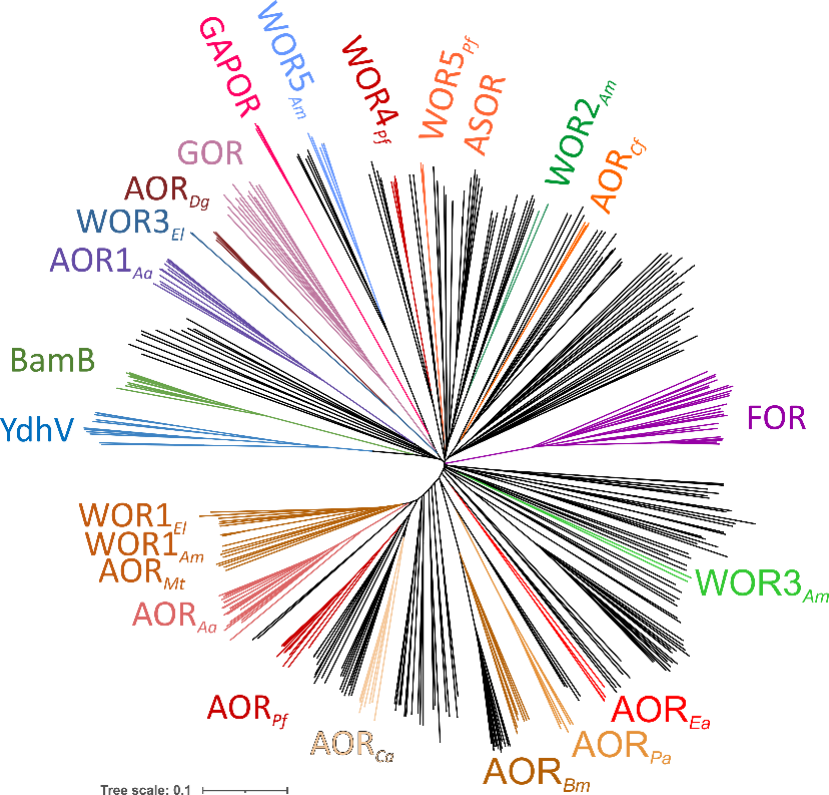
Phylogenetic tree of the AOR/WOR family based on sequences of W-co-containing subunits. The tree shows the branching pattern and relative positions of most known subcategories, including hypothetical proteins that are only known as protein sequences encoded in the genomes of *Bacteria* or *Archaea* (unlabeled black branches). The categories containing biochemically or structurally defined enzymes (or paralogues encoded in the same species) are represented by the following subcategories: AOR_*Xy*_ represent various subfamilies of AORs with broad substrate spectra from different bacteria or archaea with very similar catalytic subunits, but different quaternary structures and accessory subunits. Enzymes from *Eubacterium* and *Acetomicrobium* species are represented as WOR1–3_*El*_ and WOR1–5_*Am*_. GAPOR and GOR represent glyceraldehyde:ferredoxin oxidoreductases from *Archaea* and *Bacteria*, respectively. AOR1 is a putative second W-protein from *A. aromaticum* and related bacteria; WOR4_*Pf*_, FOR, and WOR5_*Pf*_ */ASOR* are characterized as additional W-proteins from *P. furiosus*. BamB represents the W-containing subunit of the benzoyl-CoA reductase complexes from *Geobacter* species, and YdhV represents the homologous Mo-proteins of unknown function. Sequences were aligned by Clustal omega,^13^ and the tree was constructed using iTOL^14^ (*Aa* – *Aromatoleum aromaticum*, *Pf* – *Pyroccocus furiosus*, *Ca* – *Clostridium autoethanogenes*, *Cf* – *Clostridium formicoaceticum*, *Bm* – *Brevibacillus massiliesis*, *Pa* – *Pyrobaculum aerophilum*, *Ea* – *Eubacterium acidaminophilum*, *Dg* – Desulfovibrio gigas).

## Aldehyde Oxidoreductases (AOR *Sensu Stricto*)

AOR are W-enzymes from *Archaea* or *Bacteria* that catalyze the oxidation of various aldehydes. Signature enzymes of this group are the enzymes from Pyrococcus furiosus, *Aromatoleum aromaticum*, and *Moorella thermoacetica* (AOR_*Pf*_, AOR_*Aa*_, AOR_*Mt*_), which represent different subclades. Their physiological function often seems to be the detoxification of aldehydes accumulating in the cells during the metabolism of organic compounds, as suggested by their wide substrate range and varying abundancies in cell extracts, although their occasional presence at very high abundances under some growth conditions suggests that they may exert additional, still unclear functions. For example, AOR_*Aa*_ was shown to take over the oxidation of phenylacetaldehyde in phenylalanine degradation if the enzyme otherwise dedicated to this reaction is lost.^15^ AORs catalyze the two-e^–^ oxidation of aliphatic and aromatic aldehydes (including heteroaromatic aldehydes like furfural or 2-thiophene carboxaldehyde^5,16^ ) to the respective carboxylic acids and transfer the abstracted electrons either to ferredoxin (Fd) or to NAD^+^ as electron acceptors, depending on the architectures of the respective enzyme complexes. However, at least some of these enzymes are also capable of reducing nonactivated acids to aldehydes, representing the only known occurrence of this reactivity in biochemistry. Other NAD­(P)-coupled aldehyde dehydrogenases (superfamilies cl11961 and cl49616 in the conserved protein domain database) or Mo-co containing aldehyde oxidases (superfamily cl29417), which are affiliated to the xanthine dehydrogenase family of molybdoenzymes,^17^ are mostly only able to oxidize aldehydes to acids. Some of the aldehyde dehydrogenase family members are involved in reducing organic acids to the corresponding aldehydes, but they always need prior activation of the acids to acyl-phosphates or -thioesters.^18^

The AOR-type enzymes (*senso stricto*) are divided into several subclades, whose members contain very similar catalytic subunits containing the conserved W-cofactor and Fe_4_S_4_ cluster, but differ widely in their quaternary structures and the presence or absence of additional subunits in the enzyme complexes (Figure 1). The biochemically characterized examples as well as some interesting cases of predicted AOR subtypes are presented as follows:

(i) **AOR**_***Pf***_. Aldehyde:ferredoxin oxidoreductases from *Pyrococcus* species and related *Archaea* exhibit very high optimal temperatures (>60 °C), occur as α_2_ homodimers, and require Fd as an electron acceptor. In addition to the W-*bis*-(MPT)-Mg bimetallic cofactor and the Fe_4_S_4_ cluster present in each subunit, these enzymes are distinguished by the presence of a bound Fe^2+^ ion at the subunit interface (Figure 1).^19^ This bridging Fe^2+^ is unique for this subclade, since the ligating residues Glu332 and His383 are not conserved in any other members of the family. While mostly assessed in the direction of aldehyde oxidation, their principal activity in acid reduction has been demonstrated.^20^
(ii) **AOR**_***Aa***_. AOR from the facultatively anaerobic denitrifying bacterium *A. aromaticum* (AOR_*Aa*_) is active at ambient temperature and remarkably resistant against exposure to oxygen, compared to most other AORs. It contains three different types of subunits in a large complex, and its structure was recently characterized by cryoEM and mass photometry.^21^ The AorB subunit represents the large catalytic subunit containing the conserved W-cofactor and Fe_4_S_4_-cluster, but instead of forming homooligomers, it associates with the small polyferredoxin-like AorA subunit, which adds four additional Fe_4_S_4_ clusters to each AorAB dimer. These heterodimers are stacked on top of the FAD-containing third subunit AorC, forming long filaments, which harbor a nanowire of electrically conductive Fe_4_S_4_ clusters in their center. In addition, the filaments contain diverging junctions of Fe_4_S_4_ clusters toward the W-cofactors in the catalytic AorB subunits, which branch off the side of the filament (Figure 1).^21^ This allows the electrons recovered from aldehyde oxidation to be transferred along the nanowires until they arrive at the FAD cofactor of AorC, where they can be transferred to NAD^+^. In addition, AOR_*Aa*_ was shown to use hydrogen as an electron donor to reduce either NAD^+^ or organic acids.^22^ Stacking of the AorAB protomers to a long filament appears to depend on the presence of a C-terminal extending helix in the AorA subunits, which stabilizes the structure.^21^ In addition to oxidizing a wide range of aldehydes, AOR_*Aa*_ has already been shown to efficiently reduce acids to aldehydes in several applications (see below).^22–25^

(iii) **AOR**_***Mt***_ This AOR occurs in the strictly anaerobic and thermophilic bacterium *Moorella thermoacetica* and has initially been characterized as a “carboxylic acid reductase” (CAR) for its ability to reduce acids to aldehydes with artificial low-potential electron donors.^26^ Judging from the operon structure and functional assignments of its coding genes, it also consists of three subunits sharing strong similarities to those of AOR_*Aa*_, but its actual composition is still unclear. The absence of a C-terminal extension in its AorA subunits suggests that it probably associates into an AorABC trimer, but does not form longer filaments (Figure 1).^26^
(iv) **AOR**_***Ca***_ An AOR highly similar to AOR_*Pf*_ has been characterized from the anaerobic bacterium *Clostridium autoethanogenes*, but this enzyme occurs as a monomer and should be affiliated to a separate subclade, based on the absence of the Fe^2+^ ion bridging the subunits in AOR_*Pf*_ (Figure 1).^27^ Accordingly, it uses Fd as a physiological electron acceptor, while no evidence has been found for the use of hydrogen as an electron donor. In contrast to the structures of AOR_*Pf*_ and AOR_*Aa*_, the W-cofactor of AOR_*Ca*_ appears to be either in a different redox state or in an inactivated form in the crystals (see below).^27^ Using this enzyme, a coupled pathway has been set up for alcohol production driven by CO.
(v) **WOR1**_***El***_ New members of the AOR/WOR family have recently been found in anaerobic gut bacteria like *Eubacterium* or *Acetomicrobium* species. These microbes often contain multiple genes for different W-enzymes from several clades in their genomes (three paralogues in *Eubacterium* species and five in *Acetomicrobium* species).^5^ One of these paralogues, WOR1, is encoded in species of both genera and is of particular interest because it is predicted to form a large complex with four other subunits encoded in a common operon. This complex contains a heterodimeric unit of the catalytic W-cofactor and Fe_4_S_4_-cluster containing subunit Wor1L and the polyferredoxin subunit Wor1S contributing four more Fe_4_S_4_ clusters, which are affiliated to the same phylogenetic clade as the corresponding subunits of AOR_*Mt*_ (Figure 2). However, while AOR_*Mt*_ forms a complex with an FAD-binding third subunit similar to that of AOR_Aa_, the WOR1LS protomer appears to assemble with a trimeric Wor1ABC module, which is similar to the known HydABC module present in many bifurcating enzymes.^28^ Therefore, WOR1 has been proposed to catalyze electron bifurcation, although it has only been proven so far to distribute electrons from aldehyde oxidation to Fd and NAD^+^ as electron acceptors, lacking any endergonic electron transfer step.^5^
(vi) **Further AOR orthologues closely related to AOR**_***Pf***_. Additional AOR orthologs affiliated to highly related subclades with those defined by AOR_*Pf*_ or AOR_*Aa*_ were characterized from the anaerobic bacteria *Eubacterium acidaminophilum* (AOR_*Ea*_ ^29^) and *Thermoanaerobacter* sp. strain X514 (AOR_*X514*_).^30^ Both enzymes are highly oxygen-sensitive, oxidize a broad spectrum of aldehydes and show the usual content of one W-cofactor and one Fe_4_S_4_ cluster per subunit. AOR_*Ea*_ was monomeric and active at ambient temperatures,^29^ while AOR_*X514*_ showed dimeric composition and was moderately thermophilic^30^ (Figure 1).
(vii) **AORs affiliated to distantly related clades**. Several more AOR isoenzymes have been characterized from various bacterial and archaeal species. While affiliated to various subclades in the phylogenetic tree, which are no longer closely related to AOR_*Pf*_ or AOR_*Aa*_ (Figure 2), they show surprisingly similar properties to the latter, such as the presence of a W-co and an Fe_4_S_4_ cluster and oxidation of a broad spectrum of aldehydes with viologen dyes as artificial electron acceptors. The characterized enzymes include homodimeric enzymes (AOR_*Dg*_ from Desulfovibrio gigas,^31^ AOR_*Cf*_ from *Clostridium formicoaceticum*,^32^ AOR_*Pa*_ from the hyperthermophilic denitrifier *Pyrobaculum aerophilum* ^33^), and the monomeric AOR_*Bm*_ from *Brevibacillus massiliesis*, the only aerobic species known to produce AOR^34^ (Figure 1).

In addition to the residues required for binding of the W-co and the FeS cluster, most of the listed AORs contain four conserved second shell residues (Thr243, Glu313, Tyr427, His448, AOR_*Pf*_ numbering), with conservative single substitutions only observed in AOR_*Cf*_ (Tyr replaced by His) and AOR_*Ea*_ (Thr replaced by Ser). This considerable degree of conservation is one of the reasons to implement these residues in a proposed common reaction mechanism (see below).

## Glyceraldehyde-3-phosphate Ferredoxin Oxidoreductases (GAPOR or GOR)

Glyceraldehyde-3-phosphate ferredoxin oxidoreductases occur in the AOR/WOR family as two unrelated clades, monomeric GAPOR in *Archaea*, and heterotetrameric GOR in *Bacteria* (Figure 1). GAPOR are ∼63 kDa monomeric enzymes, highly specific toward the oxidation of d-glyceraldehyde-3-phosphate to 3-phosphoglycerate.^35^ They participate in a modified glycolysis pathway of hyperthermophilic *Thermococcales* which does not involve the intermediate 1,3-bisphosphoglycerate and therefore does not couple this reaction with subsequent ATP regeneration.^36^ Like most other W-enzymes from *Thermococcales*, GAPOR uses Fd as a natural electron acceptor and can be artificially coupled with viologen dyes. Another member of the GAPOR clade was described from *Methanococcus maripaludis* as a recombinant enzyme produced in E. coli which surprisingly contained Mo and was inhibited by W.^37^ However, it remains to be seen whether Mo is also present in the native enzyme or was wrongly incorporated in E. coli. The GOR clade represents a different “non-coupled” tungsten containing glyceraldehyde-3-phosphate oxidoreductase from thermophilic fermentative *Bacteria*, such as *Caldicellulosiruptor bescii*.^38^ Interestingly, only the second shell Thr and Glu residues are conserved in the GAPOR clade, while there is no detectable similarity of the region covering the otherwise conserved Tyr and His to other family members. In contrast, all four residues are conserved in the GOR clade.

## Formaldehyde Oxidoreductases (FOR)

FOR, which was first discovered in hyperthermophilic *Archaea*, is characterized by a narrow substrate range that limits its catalytic activity to short-chain (C1–C4) aliphatic aldehydes, but the lowest K_m_ was observed for glutaric dialdehyde.^39^ This may suggest that the native substrate of FOR is not actually formaldehyde but glutaric semi- or dialdehyde. The structures of FOR from *P. furiousus* (PDB 1B25, 1B4N) revealed that the enzyme is a homotetramer where each ∼65 kDa subunit contains a W-*bis*-(MPT)-Mg bimetallic cofactor and one Fe_4_S_4_ cluster.^40^ The presence of a bound glutarate molecule in the active site of the crystals reaffirms the proposed physiological function of FOR in oxidizing glutaric semialdehyde. All four second shell residues predicted to be involved in the reaction mechanism are conserved.

## WOR4_*Pf*_

The fourth genomically encoded W-protein from *P. furiosus* (WOR4_*Pf*_) was purified by enriching W-containing fractions from cell extract, which are not associated with the three previously known W-enzymes AOR, FOR or GAPOR. WOR4_*Pf*_ turned out to be a homodimeric protein containing 1 W, 1 Ca, and 3 Fe per subunit and exhibited an EPR spectrum consistent with the presence of an Fe_3_S_4_ instead of the usual Fe_4_S_4_ cluster.^41^ However, no activity was recorded for the oxidation of any aldehydes or other potential substrates. The presence of an unusual Fe_3_S_4_ cluster in members of the WOR4_*Pf*_ clade appears to be correlated with the exchange of one of its Cys ligands to Gly, while the loss of AOR activity may also be related to the exchange of the otherwise conserved second shell Tyr residue to Trp.

## Aliphatic Sulfonate Ferredoxin Oxidoreductase (ASOR Formerly WOR5)

WOR5_*Pf*_/ASOR represents the fifth tungsten enzyme encoded in the genome of *P. furiosus* and has been characterized as a heterodimer (α_2_β_2_) consisting of a 64.8 kDa catalytic α subunit, which contains a W-*bis-*(MPT)-Mg cofactor and one Fe_4_S_4_ cluster ligated by 3 Cys and one Asp, and a small 18 kDa polyferredoxin-like β-subunit, which contains 4 additional Fe_4_S_4_ clusters (Figure 1). Like other AORs, it has been reported to oxidize a wide range of aliphatic and aromatic aldehydes with the lowest K_M_ values observed for larger compounds (e.g., 2-ethylhexanal or 2-phenylpropionaldehyde).^42,43^ It was also observed, that the expression of the enzyme increases when *P. furiosus* is exposed to a cold shock.^42^ The structures of WOR5_*Pf*_/ASOR (PDB 6X6U and 6X6O) confirmed that the enzyme not only forms a heterodimeric complex, but also revealed exciting information on its physiological role, based on a cocrystal with the bound substrate. While the electron density of the original data set did not allow to determine all tungsten ligands, it indicated the presence of an (*S*)-1-hydroxy-1-butylsulfonate in the active site as an apparent substrate mimic, which was also corroborated by analyzing the anomalous signal for the close-by tungsten. This prompted biochemical assays and cocrystallization attempts with taurine as a similar sulfonate, which occurs naturally as a compatible solute. WOR5_*Pf*_/ASOR indeed showed clear taurine binding into its active site and exhibited significant activity in converting taurine to glycine betaine and sulfite. Since this reaction represents a four-e^–^ oxidation, WOR5_*Pf*_/ASOR was proposed to initially catalyze the oxidation of taurine to betaine aldehyde and sulfite, followed by further oxidizing the aldehyde to the acid in glycine betaine.^43^ The second step represents the same type of reaction observed in the other AOR isoenzymes, while the initial step is a new quality in WOR5_*Pf*_/ASOR, which has therefore been renamed to “aliphatic sulfonate ferredoxin oxidoreductase”. The gain of this new function may be correlated with changes in the second shell residues of the active site: the otherwise conserved Thr is lost and Tyr is replaced by His (as also seen in AOR_*Cf*_).

## Structure of the Active Sites and Proposed Catalytic Mechanisms of AOR/WOR Family Members

### AOR

The best studied AOR is the one from *P. furiosus*, a hyperthermophilic archaeum growing optimally at 100 °C by a fermentative type metabolism, producing H_2_ and CO_2_ as main end products. AOR_*Pf*_ is highly thermostable, but also strongly sensitive to inactivation by O_2_. It was the first representative of tungsten or molybdenum enzymes to be structurally characterized^19^ (PDB 1AOR), and revealed the coordination of the tungsten ion by two metallopterins, each bound to the metal with two dithiolene sulfur atoms in a distorted square pyramid geometry. In the originally deposited structure, the authors did not refine additional ligands of the W ion, although evident electron density was present. This was recently amended by reevaluation of the original electron density data, combined with QM:MM modeling of potential candidate structures by Winiarska et al., who proposed that two additional oxido ligands coordinate the W­(VI) metal center^21^ (Figure 3). The same structure of the cofactor was proposed for the highly similar large subunit of the AOR_*Aa*_ which was resolved by cryoEM (PDB 8C0Z) and showed a significantly lower resolution than the structure of AOR_*Pf*_. Finally, a recently published high-resolution structure of AOR from *Clostridium autoethanogenum* (PDB 9G7J) also corroborates the presence of two oxygen ligands on the tungsten ion, although the W–O distances suggest the presence of OH rather than oxido groups, which may either indicate the presence of a reduced state of the W-co (e.g., due to photoreduction by X-ray irradiation) or some inactivated form of the enzyme.^27^

**Figure 3:**
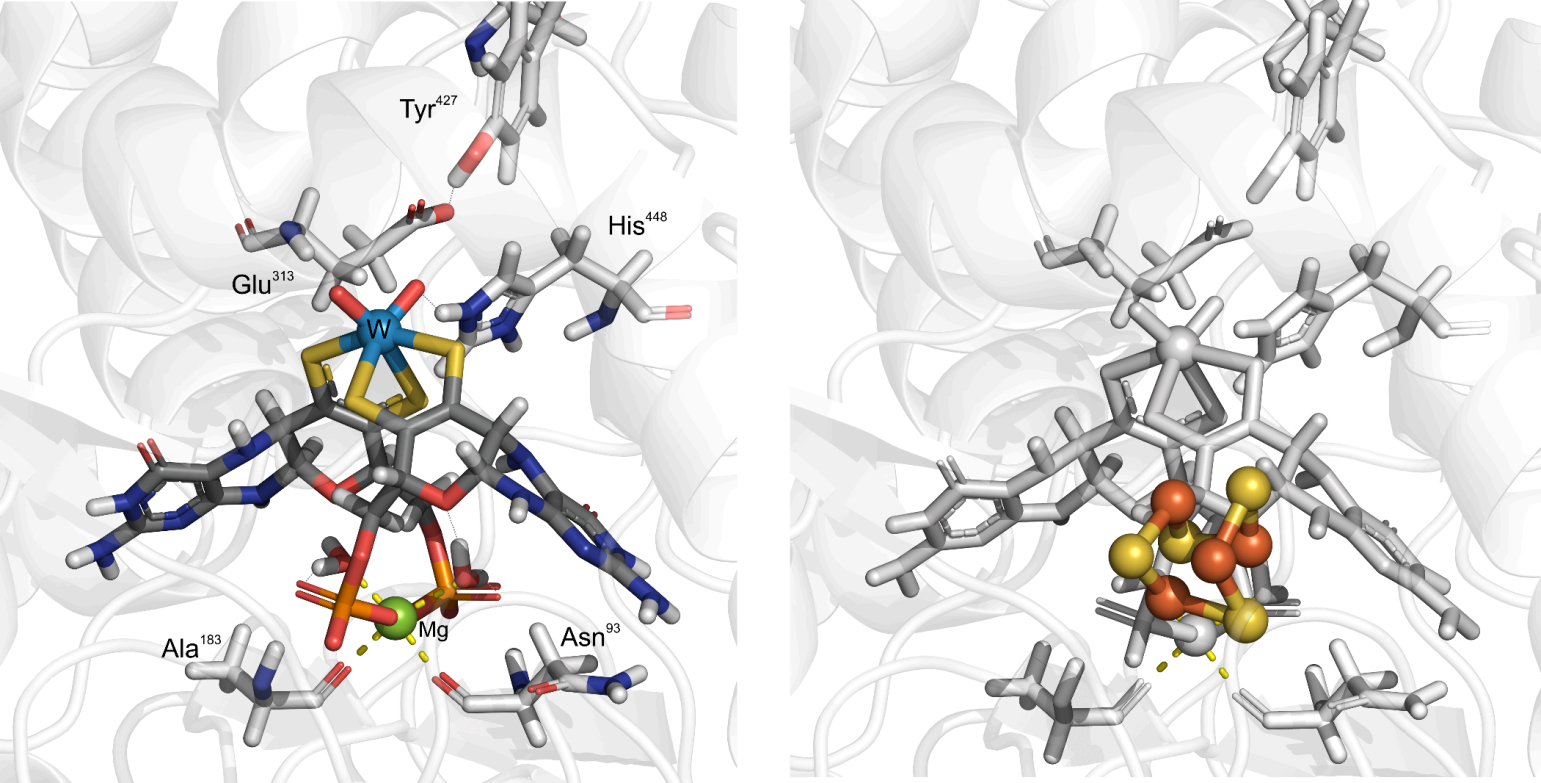
Active site of AOR_*Pf*_ after reinterpretation of electron density and representing the main conserved second shell residues.^21^ H-bonds shown by a gray dotted line. The coordination bonds of Mg shown as a yellow dashed line. The right panel presents the position of the Fe_4_S_4_ cluster with respect to the cofactor.

The two MPT ligands are linked through a Mg^2+^ ion ligated to their phosphate groups in all known AOR structures. The Mg^2+^ is also coordinated by carboxyl groups from the main chain of the enzyme (Asn93 and Ala183 in AOR_*Pf*_) and two water ligands, attaining an octahedral coordination geometry. The Mg^2+^ seems to exhibit a structural role by keeping both MPT ligands in a less extended conformation and potentially modulates the redox potential of the W-cofactor. While no amino acid residue is directly bound to the W ion, the universally conserved residues Glu313, His448 and Tyr427 are located in its second coordination sphere in the known AORs and may provide functional groups for acid–base catalysis. There are also two additional highly conserved tyrosines (Tyr312, Tyr452) in the active site, which may be involved in the proton-relay system as well as Thr243 (sometimes substituted to Ser). The latter may form an H-bond with one of the W=O ligands or with the carboxylic group of Glu311, another highly conserved residue flanking the W-co from the opposite side of the substrate cavity. All these highly conserved residues surrounding the W-co are able to act as general acid/base catalysts assisting the tungsten cofactor in both redox and acid/base processes.

### FOR

Two FOR_*Pf*_ structures have been solved i.e., one with a glutarate bound to the active site (PDB 1B4N) and the other with one oxygen ligand positioned at a quite strange angle, which indicates that some other ligand may be present, but not revealed by the refinement (PDB 1B25; Figure 4).^40^

**Figure 4:**
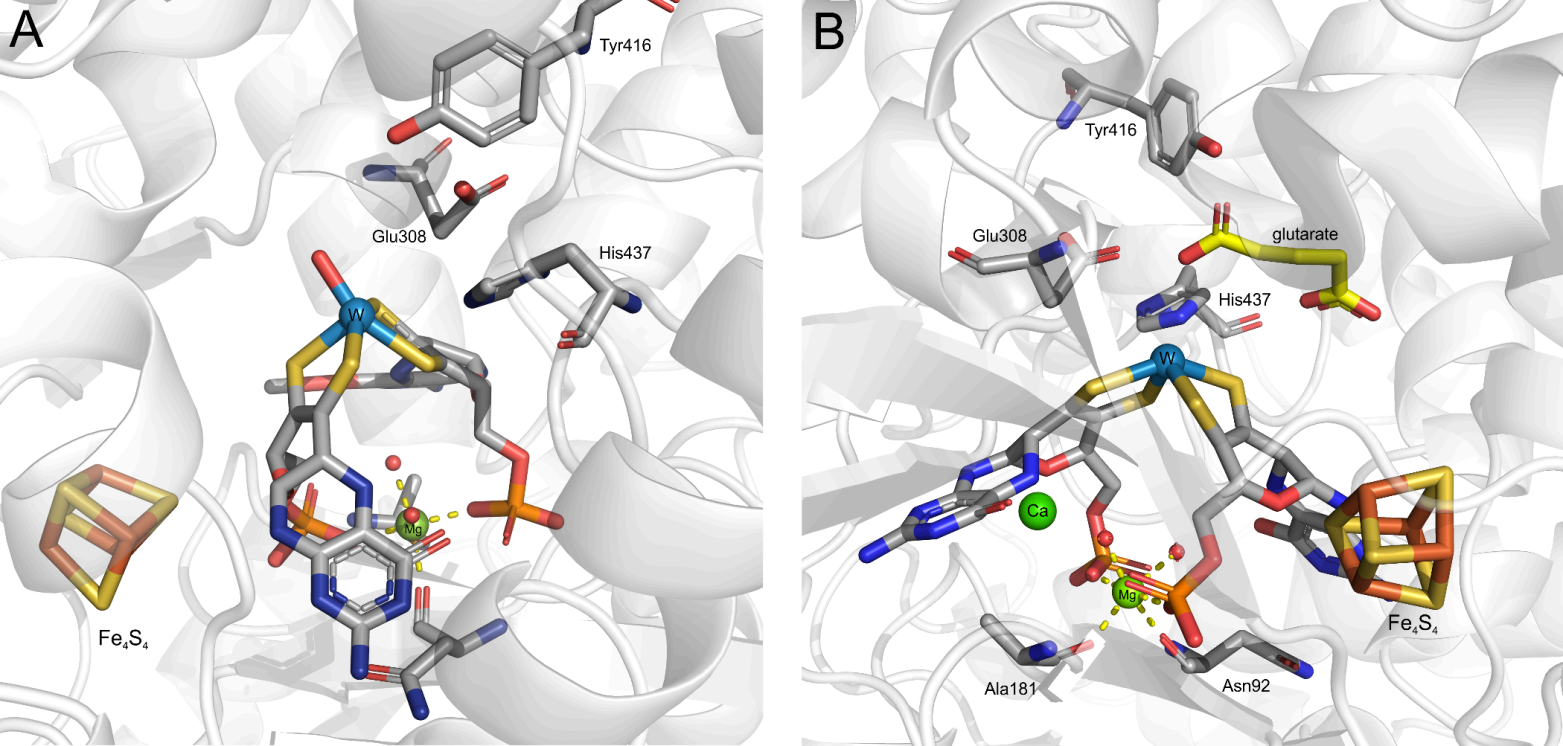
Active site of formaldehyde oxidoreductases: A) as prepared, B) in complex with glutarate. The conserved second shell residues are indicated.

The crystal structure revealed that the W ion, besides being coordinated by four pterin dithiolene sulfur atoms, has only one oxygen-type ligand at a distance of 2.1 Å with no additional ligand observed. The odd geometry of the cofactor (Figure 4A) and available EXAFS results^44^ suggest the presence of yet another ligand. The authors indicated that the lack of additional ligand may be due to the heterogeneity of the tungsten oxidation state. The enzyme also contains a binding site for one calcium ion (Figure 4B) which interacts with the carbonyl atom of one of the metallopterins and protein residues and most probably has a structural role.

## Proposed Reaction Mechanisms for AOR and FOR

At the moment, two types of mechanistic hypotheses have been proposed to explain the catalytic activity of the aldehyde oxidizing or acid reducing enzymes (i.e., AOR and FOR). The first type represents a first coordination sphere mechanism, which assumes direct binding of the aldehyde to the W­(VI)­O­(MPT)_2_. Two versions of this mechanism were discussed in the literature. The first scheme^45^ (Figure 5) proposes the unprotonated conserved Glu residue to activate a water molecule, which then conducts a nucleophilic attack on the carbonyl atom of the aldehyde bound to W-co. Potential interactions of the aldehyde with W-co and hydrogen bonding with the conserved Tyr may increase the positive partial charge of the carbonyl carbon enough to allow the transfer of a hydride equivalent to the W=O group, formally reducing the cofactor to the W­(IV) state. An OH ligand would be formed concomitantly on the W ion. After transferring the proton from the formed acid product to the Glu, the reaction cycle ends with the acid bound to the reduced W­(IV)–OH cofactor. After the product is released and replaced by water, the W-co then undergoes deprotonation and reoxidation in two one-e^–^ steps via the close-by Fe_4_S_4_ cluster.

**Figure 5:**
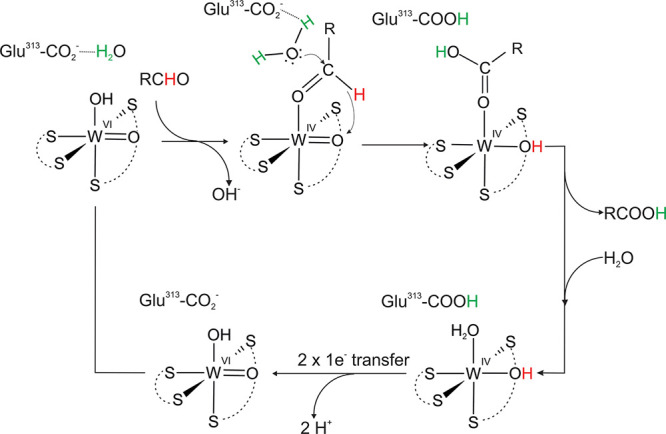
First coordination sphere mechanism for AOR proposed by Bevers et al.^45^

A second first sphere mechanism (Figure 6) was based on modeling study conducted for FOR.^46^ Both first and second shell mechanistic variants were considered, but only the former was associated with kinetically accessible barriers while the second sphere mechanism showed an prohibitively high barrier for hydride transfer. In the first sphere mechanism, the aldehyde is binding to the oxidized W–co [W­(VI)­O­(MPT)_2_] as proposed for AOR_*Pf*_, but then one of the oxido ligands of the W­(VI) conducts a nucleophilic attack on the carbonyl C atom, which results in converting the bound aldehyde to a deprotonated geminal diol intermediate bound to the W ion in a bidentate mode. Next, the H^+^ is transferred to Glu308 (FOR_*Pf*_ numbering) with the concomitant transfer of two electrons to the W­(VI), which results in its reduction to W­(IV). The deprotonated product is still bound in a bidentate mode to the reduced cofactor, before being released as acid with two incoming water molecules.

**Figure 6:**
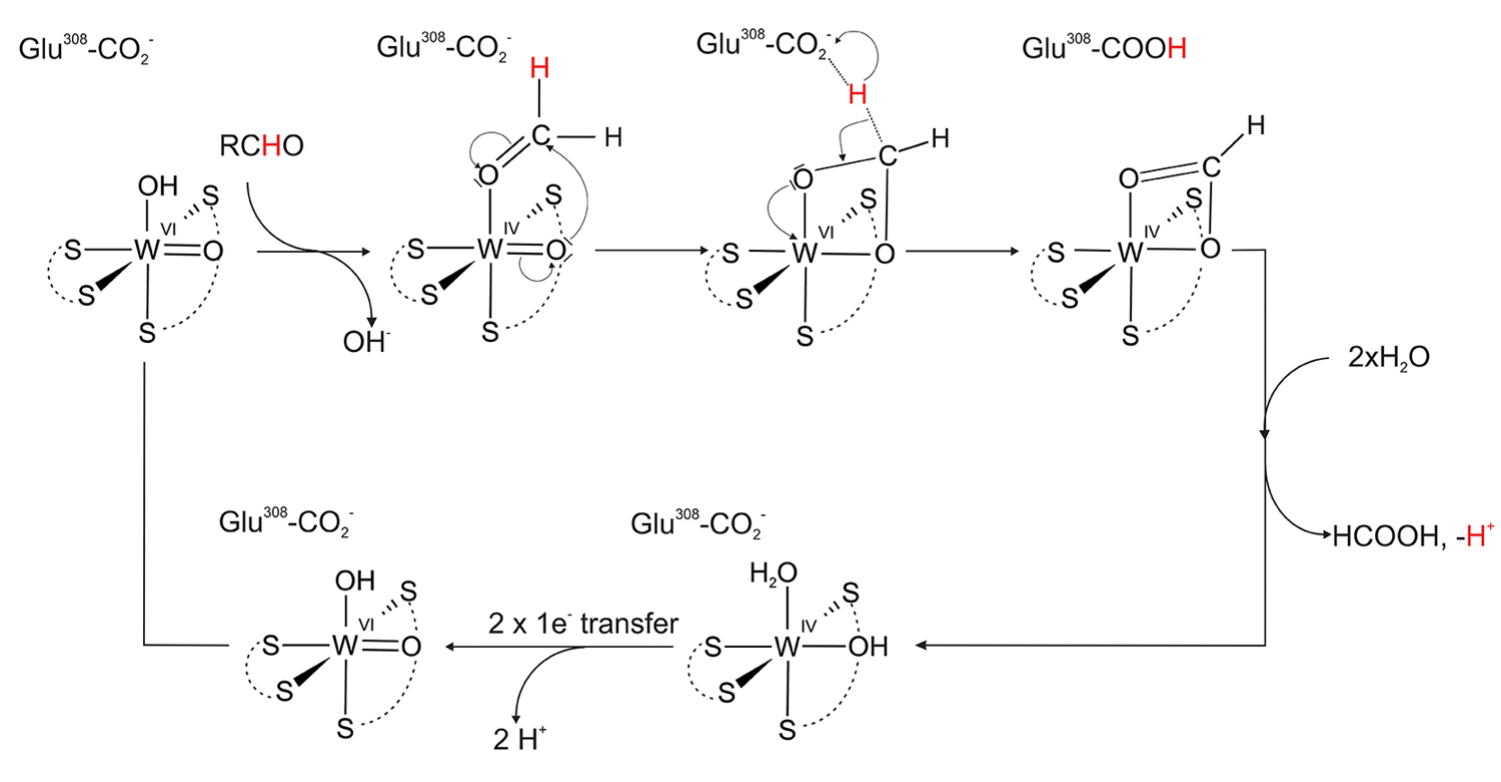
First coordination sphere mechanism for FOR_*Pf*_ proposed by.^46^ Note that Glu308 is equivalent to Glu313 in AOR_*Pf*_.

Both original mechanisms do not account for the presence of two oxygen ligands at the W­(VI)-co although in a recent review by Das et al.^7^ it was proposed that the starting cofactor form is W­(VI)­O­(OH)­(MPT)_2_ and the ligand exchange between a hydroxide and the aldehyde would be a first step of the mechanism.

Also, while these mechanisms can explain the mechanism of aldehyde oxidation, it is harder to imagine the same mechanism running backward for acid reduction. Because of the apparent reversibility of various AORs and based on the reinterpretation of the 1AOR crystal structure which implied two oxygen-type ligands bound to the W atom as well as new EXAFS data suggesting a W­(IV)­O­(OH)­(MPT)_2_ form of the reduced cofactor (data not shown), we have made yet another proposal of a second sphere mechanism.^21^ To overcome the high barrier of hydride transfer calculated by Liao et al.,^46^ we propose the formation of the hydrated form of the aldehyde (i.e., a geminal diol) as a first step in the active site (Figure 7). As the formation of a hydrated aldehyde in solution is catalyzed by either acidic or basic conditions (i.e., protonation of the oxido group or attack of hydroxide at the carbonyl carbon of the aldehyde), we propose that this process may be assisted by general acid–base catalysis. A water molecule activated by one of the W=O ligands would attack the carbonyl carbon atom with concomitant H^+^ transfer to the carbonyl oxygen atom. The positioning of the aldehyde would be facilitated by H-bonding with Tyr427 (AOR_*Pf*_ numbering). The transfer of the proton from the geminal diol to Glu313 would increase the negative charge at the carbonyl carbon atom and facilitate hydride transfer to the W-co, forming the reduced form of the cofactor [W­(IV)­(O)­(OH)­(MPT)_2_] and a protonated acid. This process may be assisted by H-bonds formed between the reagents, His448 and Thr243 (which also likely forms an H-bond with the other W=O ligand). Removal of the protons from the W-co active site would be assisted by the Glu313-His448 proton relay (extended by Tyr312 and Tyr452), while the reoxidation of the W-co would proceed in two one-e^–^ steps as in the other proposed mechanisms.

**Figure 7:**
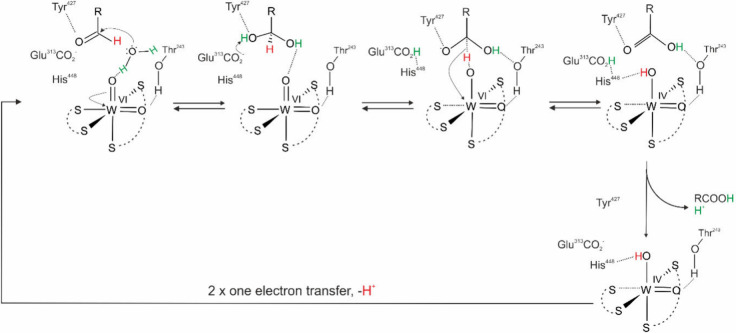
A second coordination sphere mechanism for AOR proposed by ref 21 and extended in this work.

Another unexpected feature of AOR was recently discovered while working on AOR_*Aa*_. The enzyme clearly uses H_2_ as an electron donor for reducing either NAD^+^ to NADH or acids to aldehydes, thus exhibiting hydrogenase activity. Although no similar activity was observed for other members of the AOR/WOR family so far, with at least one case tested,^27^ this observation would fit better with a second shell mechanism than those previously proposed. To date, no mechanism that could explain the observed hydrogenase activity of AOR_*Aa*_ was proposed in the literature with the exception of model complexes (see below). Notably, the activity of AOR_*Aa*_ with H_2_ as an electron donor is only 10-fold lower compared to its activity with benzaldehyde,^22^ while it does not compete with the rate of actual hydrogenases. Still, the reactivity of AOR_*Aa*_ with H_2_ may become highly beneficial for potential applications, allowing the simultaneous recycling of NADH and the reduction of acids by an economically and ecologically convenient reductant. Both applications have been recently demonstrated in a cell-free cascade system (see below).

## Structure and Proposed Mechanism for Aliphatic Acylsulfonate Ferredoxin Oxidoreductase (WOR5_*Pf*_/ASOR)

WOR5_*Pf*_/ASOR catalyzes sequential two-step oxidations of taurine to glycine betaine, where the sulfonate group is eliminated in the first step, producing betaine aldehyde, which is oxidized to glycine betaine in the next step.^43^ While the second step reflects the usual aldehyde oxidation activity of all AOR, the initial reaction appears different enough to warrant special consideration. This process is apparently catalyzed by the same active center also catalyzing aldehyde oxidation and the residues involved are basically the same as in the other AOR subfamilies, except for the lack of the otherwise conserved Thr or Ser and an exchange of the conserved second shell Tyr residue to a His (His 446 in Figure 8).

**Figure 8:**
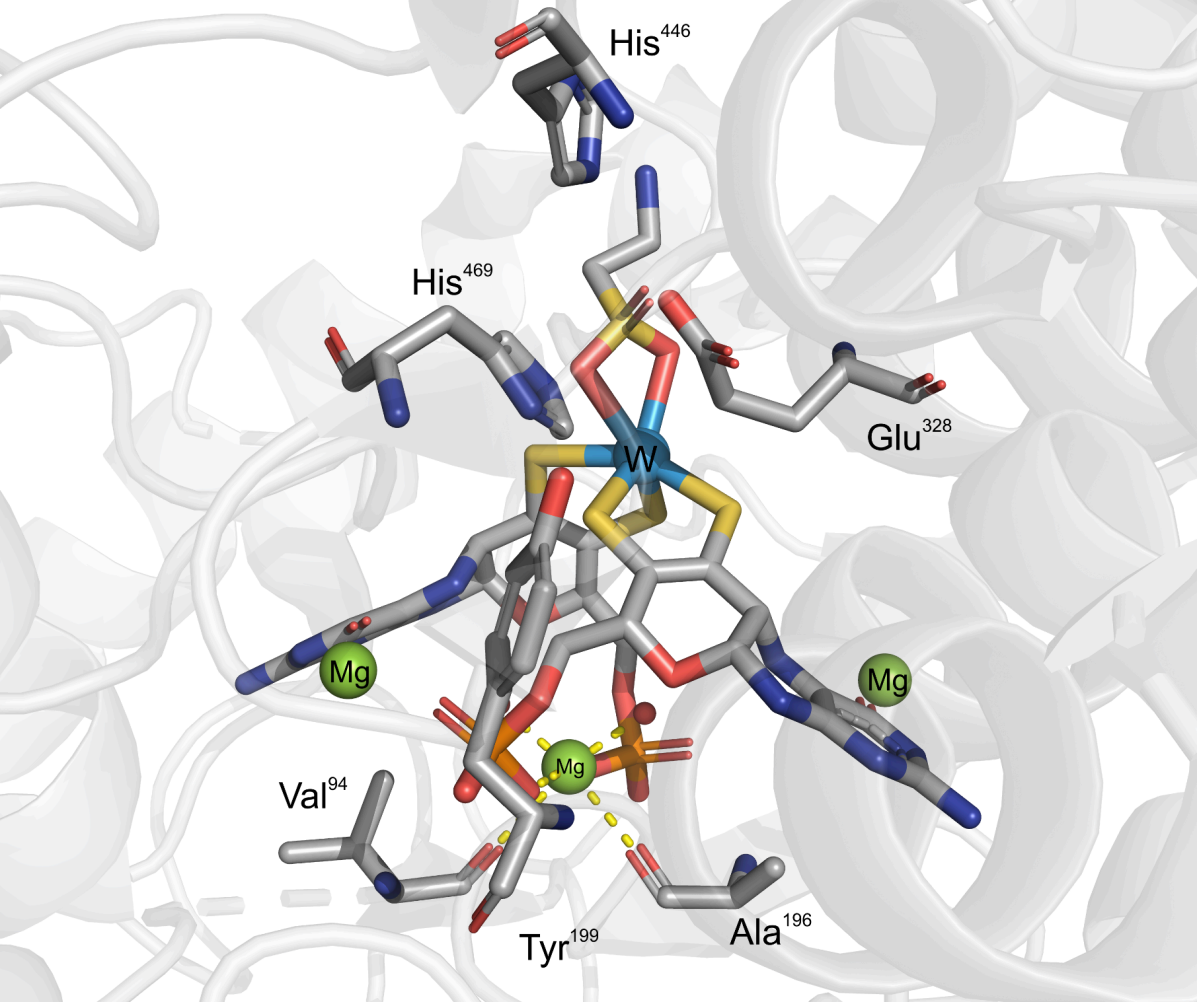
Active site of WOR5_*Pf*_/ASOR containing the W-*bis-*(MPT)-Mg cofactor with a bidentate taurine ligand bound to tungsten. The Mg^2+^ bound by the phosphate groups of the metallopterins is additionally coordinated by two water molecules and backbone carbonyl atoms from Val93 and Ala196. Two additional Mg^2+^ ions are present in the vicinity of the carbonyl groups of the metallopterin rings, reminiscent of the Ca^2+^ ion in FOR_*Pf*_. The second shell residues of Tyr199, Glu328, His446, and His469 are expected to participate in catalysis. Tyr199 forms a hydrogen bond with His469 and is unique in the WOR5_*Pf*_/ASOR clade, replacing an otherwise conserved Arg.

The second shell amino acids of WOR5_*Pf*_/ASOR are expected to support the same general acid/base catalytic steps involved in any of the proposed AOR mechanisms (Figure 9), supporting the second step of oxidizing betaine aldehyde to the betaine. The first step involves the attack on taurine, which is harder to formulate. The bidentate binding of the sulfonic acid group at the W ion observed in the structure has led to proposing an initial attack of the oxidized W­(VI) via its oxido ligand at the sulfonate group, which is activated for nucleophilic attack by coordination to the W­(VI) ion and H-bonding with His469. The formation of a transient hypervalent sulfonate then leads to the repositioning of one of the hydroxy groups from the sulfonate to the neighboring C1-atom of taurine. This step could be facilitated by abstracting the H^+^ from the C1 atom via His446 and by protonating the negatively charged oxygen of the sulfonic group via His469. After regrouping, sulfite would be released, leaving betaine aldehyde bound to the reduced W­(IV) in W-co.^43^ The reduced W-co has to be reconstituted to the catalytically active oxidized form by coordinating a water molecule followed by two one-e^–^ oxidation steps coupled with H^+^transfers. This leads to the formation of W­(VI)­(O)­(aldehyde)­(MPT)_2_ form of the cofactor. The final step is proposed to proceed along the mechanism calculated for FOR_*Pf*_.^46^

**Figure 9:**
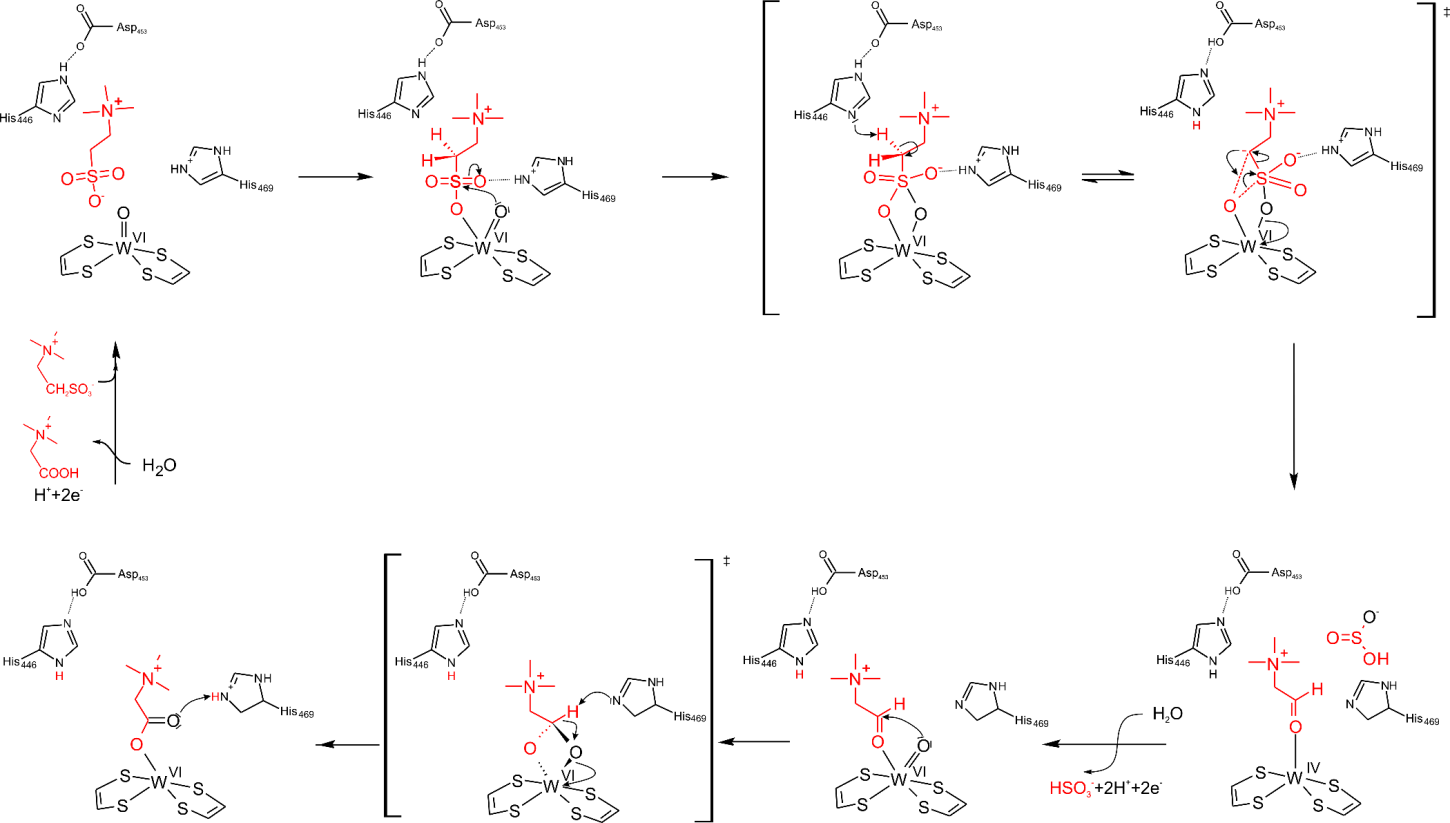
A four-e^–^ redox mechanism proposed for aliphatic sulfonate ferredoxin oxidoreductase (WOR5_*Pf*_/ASOR). Figure adapted with permission from ref 43. Copyright 2022, The Author(s), under exclusive licence to Society for Biological Inorganic Chemistry (SBIC).

We propose instead that the species seen in the structure probably represents a stable dead-end situation present in the crystals. The active form of the cofactor would be W­(VI)­(OH)­O­(MPT)_2_ and the catalytically competent ligation of the substrate likely occurs only via one of the sulfonate oxygens, after replacing the exchangeable OH^–^ ligand (Figure 10). This mode of binding localizes the C1 atom of taurine in the second ligand shell of the W ion. One of the acid–base catalysts (e.g., the unique His446, potentially assisted by the released OH^–^ from W-co) may then extract an H^+^ from C1. The resulting carbanion is stabilized by mesomeric interaction with the sulfonate group and may be favored by partial protonation of the negative charges of the oxygen residues (e.g., by His469). The (partial) C=S double bond in the transition state should be sensitive to attack by a hydroxyl ion, which leads to direct hydroxylation of the bound substrate with simultaneous transfer of two electrons to the W-co via the bound sulfonate group. The result is a hydroxyalkylsulfonate derivative of taurine bound to the reduced W–co. Since hydroxyalkylsulfonates are in equilibrium with the corresponding aldehydes and sulfite, we expect spontaneous cleavage to betaine aldehyde and sulfite, which should be pulled forward by the subsequent oxidation of the aldehyde. Oxidation of the aldehyde would proceed along the same mechanism as proposed by us for AOR, i.e. after the formation of hydrated geminal diol by H^–^ transfer to the W­(VI), either through the bound sulfite ligand (green pathway in Figure 10) or through the remaining W=O ligand. This would be followed by deprotonation/protonation steps and release of both products. The catalytic cycle would be closed by the coordination of a water ligand to W­(IV) and reoxidation to W­(VI) in two one-e^–^ steps coupled with H^+^ transfers.

**Figure 10:**
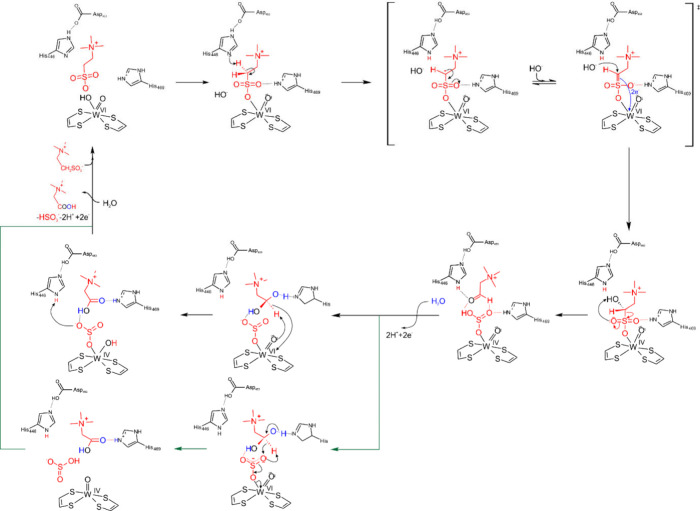
Alternate mechanism proposed for WOR5_*Pf*_/ASOR. The green arrow indicates a possible mechanistic variant of aldehyde oxidation by hydride transfer mediated via the bound sulfite ligand to tungsten ion.

## Structure and Catalytic Mechanism of Class II Benzoyl-CoA Reductases

One of the subclades of the AOR/WOR family is represented by the catalytic subunits of class II benzoyl-CoA reductases (BCR; Figure 2). Anaerobic degradation pathways of aromatic compounds mostly start with converting the original substrate to benzoyl-CoA as the central intermediate, which is then reduced to a nonaromatic cyclohexa-1,5-diene-carbonyl-CoA intermediate as a common conserved key reaction of the respective pathways.^47^ Since very low redox potentials are required for the reduction of the aromatic ring (*ca*. −622 mV for benzoyl-CoA), this step is catalyzed by enzymes with unusual biochemical properties. Depending on the microbial species involved, two unrelated classes of benzoyl-CoA reductases are known. Class I BCRs occur mostly in facultative anaerobic denitrifying bacteria, contain several catalytic FeS clusters, and depend on ATP hydrolysis to sufficiently lower the redox potential in the active site to afford aromatic ring reduction.^48^ In contrast, the ATP-independent BCRs of class II occur in strict anaerobes, typically sulfate- or ferric iron-reducing bacteria, and consist of subunits affiliated to the AOR/WOR family (BamB) and 7 additional subunits containing metallo- and flavin-cofactors (BamC-I; Figure 1), which form large complexes of about 1 MDa.^49^ Like most other members of the AOR/WOR family, the BamB subunit carries a Fe_4_S_4_ cluster and a W-cofactor, which represents the active site for benzoyl-CoA reduction. The Bam complex is believed to accomplish an endergonic electron transfer from Fd (*E*°′ = −500 mV) to the W-co of BamB (*E*°′ = −650 mV) by electron bifurcation reactions. This involves the simultaneous transfer of electrons from the starting redox potential of the electron donor (−500 mV for Fd) to acceptors with lower (−650 mV for W-co) and higher potentials (−320 mV for NADH, −70 mV for menaquinone). Thus, the endergonic process of lowering the redox potential to that of the W-co is empowered by coupling it to exergonic electron transfer processes to acceptors with higher potentials. The BCR complex contains putative output sites for coupling with either NAD^+^ or menaquinone, prompting a proposal that it may actually perform double electron bifurcation reactions toward NAD^+^ and menaquinone as a high-potential electron acceptors to establish the very low potential needed at the W-co (Figure 1).^50^

A partial complex representing a (BamBC)_2_ heterotetramer of the catalytic W-containing subunit and a polyferredoxin subunit with three Fe_4_S_4_ clusters was found to detach from the holo complex and was purified and crystallized to obtain a structure (PDB 4Z3Z, 4Z40).^51^ The structure shows that the small polyferredoxin subunits interact with each other, while each binds one of the large catalytic subunits, which do not interact with each other (Figure 11). The two W-co of the large subunits are connected by a chain of 8 Fe_4_S_4_ clusters contributed by all of the subunits, which are spaced closely enough to ensure electron transfer via quantum-mechanic tunneling. It is believed that the chain of FeS-clusters continues into the rest of the protein complex in the holoenzyme state (Figure 1). Therefore, the chain of FeS-clusters in the BCR complex exhibits a unique geometry, which differs from that of the FeS cluster arrays present in some other members of the AOR/WOR family containing polyferredoxin subunits (such as AOR_*Aa*_ or WOR5_*Pf*_/ASOR).

**Figure 11:**
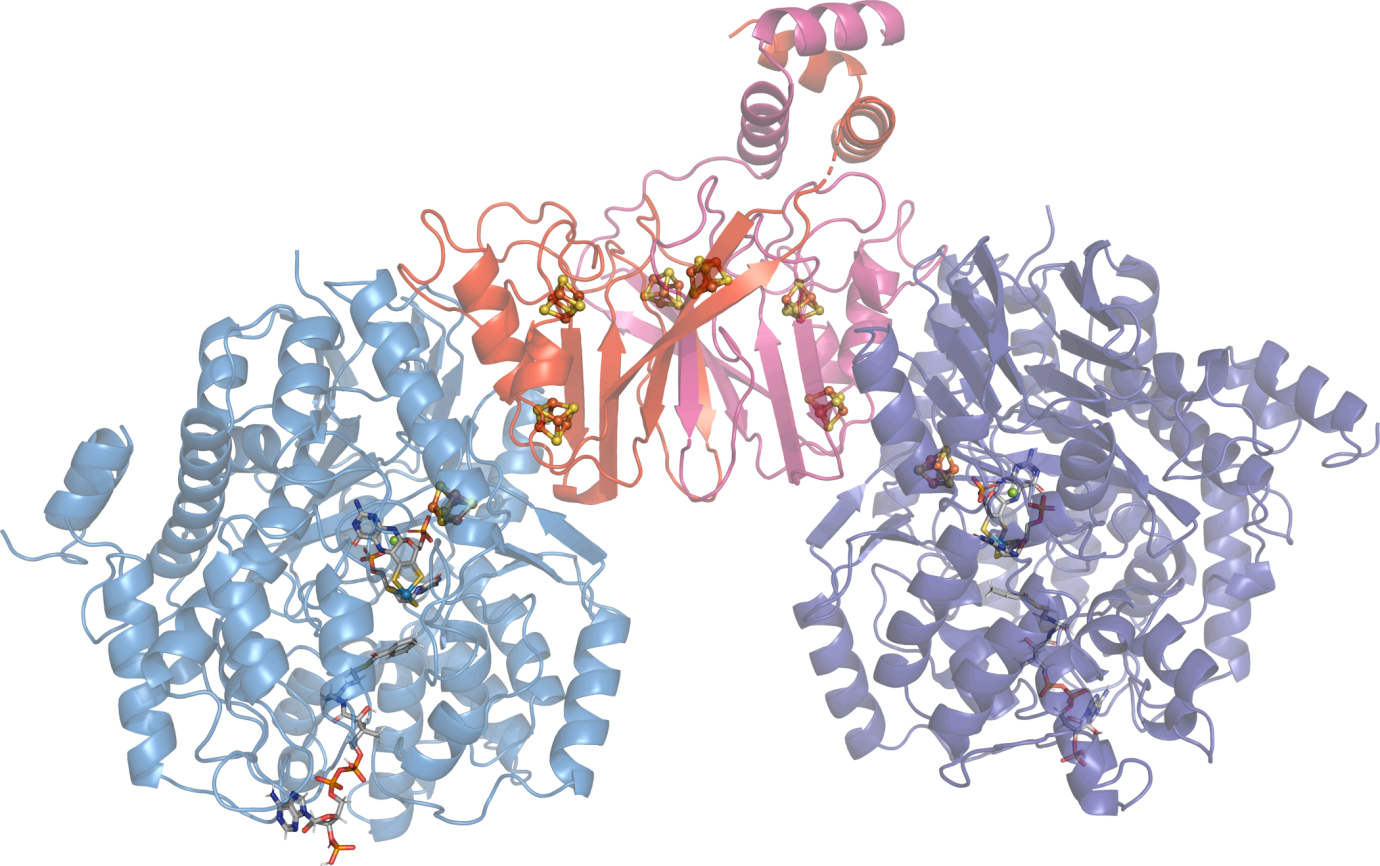
(BamBC)_2_ complex from *Geobacter metallireducens*.

The structure of the active center reveals the presence of a W-*bis*-MPT cofactor highly reminiscent to that of the other members of the AOR/WOR family, including the bridging Mg^2+^ joining the phosphates of the two metallopterins. However, in contrast to all other AOR/WOR members (except the YdhV clade), BCR contains Cys322 as a protein-based ligand to the W ion, which is conserved in all predicted BamB (and YdhV) sequences, but absent in all other AOR/WOR family members. In further contrast, BamB does not contain the potential second-shell ligands conserved in most members of the family exhibiting aldehyde oxidoreductase activity, emphasizing the different reaction catalyzed. In addition to the four dithiolene- and the Cys-based sulfur ligands, the W contains a sixth ligand, which has been identified as water. The presence of these ligands shields the W ion from any potential interactions in the first binding shell. In addition, the structure of substrate-free BamB revealed a Zn^2+^ ion ligated in the active site by the invariant residues Glu251, His 255, Glu257 and His260 at a distance of 11.5 A from the W. Upon substrate binding, the Zn-binding site is restructured, Zn^2+^ is released and the amino acids involved in Zn^2+^ binding serve as proton transfer conduit toward the active site, allowing the proton-coupled electron transfer (PCET) from the reduced W-cofactor to bound benzoyl-CoA while excluding water from entering the active site.^49,51^ The bound benzoyl-CoA is located in a largely hydrophobic pocket on top of the W-cofactor, which only includes His260 and Glu251 of the former Zn^2+^-binding motif as hydrophilic and potentially proton-donating residues. This functionality is also indicated by their positions close to the C3 and C4 atoms of the aromatic ring, which are to be reduced and protonated by the enzyme. The catalytic mechanism has been modeled by QM:MM (Figure 12), indicating that it occurs entirely in a second shell process, which is initiated by benzoyl-CoA binding and protonation of His260. This is followed by formally transferring a hydrogen atom from the water ligand of the W­(IV) ion to C4 of the aromatic ring, leading to a partially reduced radical derivative of benzoyl-CoA and a reorganized W­(V) center. Finally, a second electron is transferred from the W-cofactor to the radical intermediate, which simultaneously takes over a proton from His260, yielding the reduced cyclo-1,5-diene ring of the reduced product. Interestingly, the modeling studies suggested that the transfer of the second electron does not yield a cofactor with an oxidized W­(VI), but is producing a delocalized radical in the organic metallopterin ligands. Anyway, after the product is released, the oxidized W-cofactor needs to be reduced again in two one-electron steps to the W­(IV) starting state.^52^

**Figure 12:**
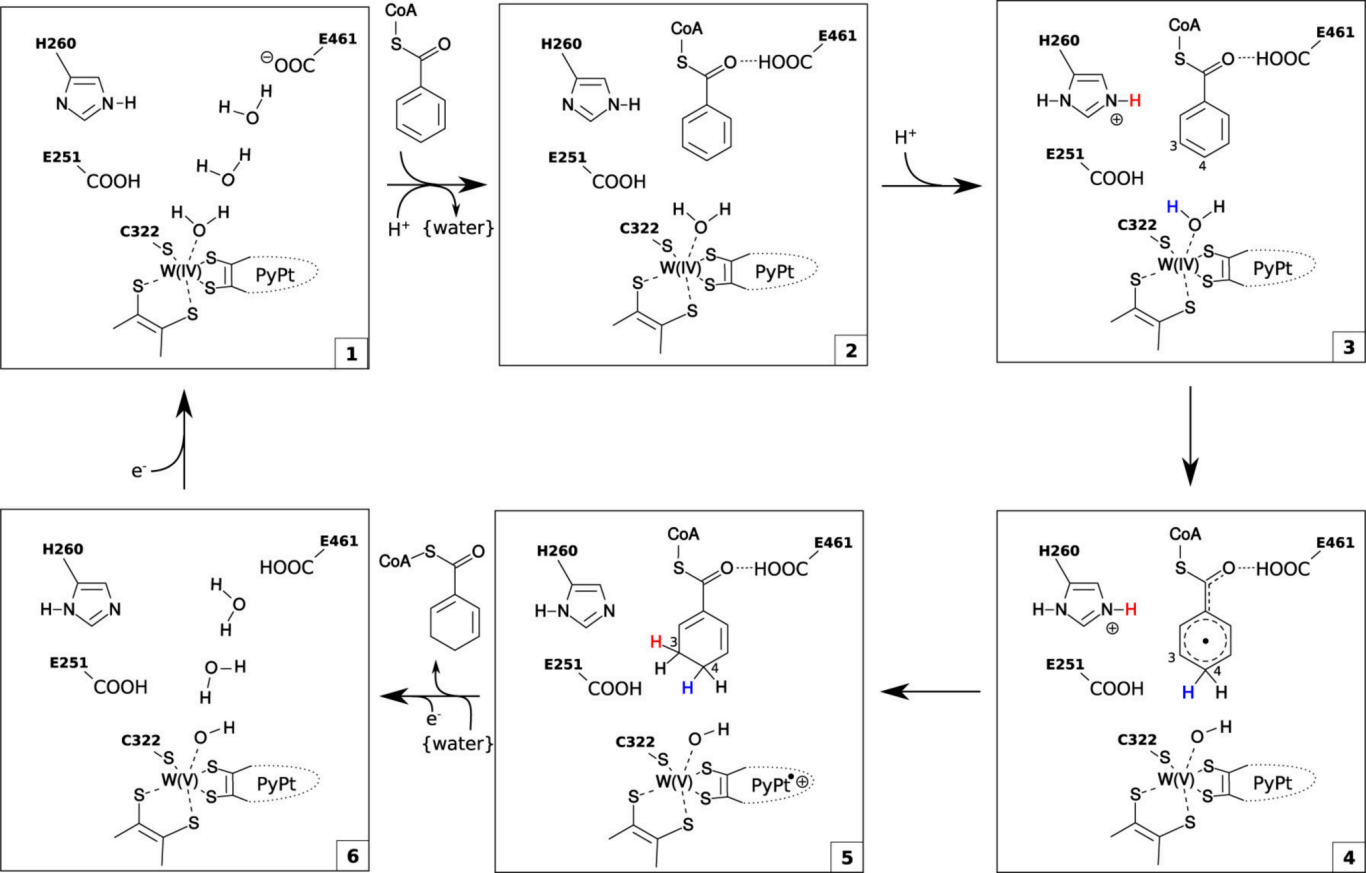
Catalytic cycle of radical benzoyl-CoA reduction by BamB proposed by Culka et al.^52^ Reproduced from Culka, M., Huwiler, S. G., Boll, M., and Ullmann, G. M. (2017), *J. Am. Chem. Soc. 139*, 14488–14500.^52^ Copyright 2017 American Chemical Society.

## DMSOR Family

### Structure and Catalytic Mechanism of Acetylene Hydratase (AH)

The last obligately W-dependent enzyme with considerable information on its structure–function relation is acetylene hydratase (AH) from the strictly anaerobic acetylene-degrading bacterium *Syntrophotalea* (formerly *Pelobacter*) *acetylenica*. In contrast to the previously mentioned proteins, AH is a member of the dimethyl sulfoxide reductase (DSMOR) family of molybdo- or tungstoenzymes,^1,49^ where it forms a separate subfamily with similar proteins, which have not been biochemically characterized so far. AH is a monomeric enzyme of 83 kDa (730 amino acids) and contains a W-*bis*-metallopterin guanosine dinucleotide (MGD) cofactor closely spaced to a Fe_4_S_4_ cluster. In contrast to the presence of these redox-active cofactors, the enzyme appears to catalyze a nonredox reaction, namely the hydration of acetylene to vinyl alcohol, which then tautomerizes to acetaldehyde. The enzyme is highly O_2_-sensitive and needs to be reduced to the W­(IV) state to be active. An X-ray structural study (PDB 2E7Z) showed that the W is bound by the dithiolenes of the two MGD cofactors and Cys141, which is strictly conserved in the other members of the AH subfamily. A sixth ligand of the W ion has been identified as a tightly bound water, similar to the W-co ligation of BamB.^53^ The Fe_4_S_4_ cluster is ligated by an N-terminal Cys motif (Cys9, 12, 16, and 46) usually conserved in the DMSOR family members, with Cys12 sharing a bridging water with one of the MGD cofactors. This Cys motif also covers Asp13, another highly conserved active site residue-specific for the members of the AH subfamily, which approaches the bound water ligand of the W ion from above the bound cofactor in the second binding shell, forming a tight H-bond. Asp13 can be mutated to Glu without causing severe effects, but enzyme activity is completely lost when is exchanged for Ala.^49^ The structure also shows the presence of a unique tunnel toward the active site, which differs from the access paths usually found in other members of the DMSOR family.

### Mechanistic Proposals for Acetylene Hydratase

Many attempts have been made to calculate a theoretical quantum-chemical model of the reaction mechanism of AH. Early DFT-based models assumed a replacement of the water ligand of the reduced W­(IV) ion by binding acetylene in the first ligand sphere. The interactions between acetylene and W­(IV) would occur via the π-electrons of the triple bond, leading to polarization and enhanced reactivity of the bound acetylene (Figure 13A). The reaction is then initiated by deprotonation of a water molecule by the conserved Asp13, producing a reactive hydroxyl ion, which adds to one of the C atoms of the bound acetylene, leading to a bound vinyl alcohol after protonation of the other C atom. At this state, the binding mode of the intermediate is proposed to reorient, resulting in vinyl alcohol binding to the W­(IV) ion via the hydroxy group, followed by tautomerization to bound acetaldehyde, which is again initiated by H^+^ transfer from Asp13 to the methylene group of the vinyl alcohol (Figure 13A). The highest barriers of this mechanism were calculated for the initial water addition at acetylene (70.7 kJ/mol) and for the final tautomerization step (59 kJ/mol).^54^

**Figure 13:**
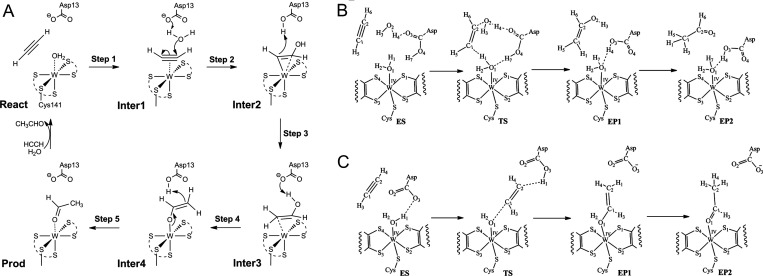
Proposed mechanisms for the catalytic activity of acetylene hydratase. A. First shell mechanism proposed by Liao et al.^54^ B and C. Second shell and first shell mechanisms proposed by Habib et al.^55^ Section B and C reproduced from Habib, U., Riaz, M., and Hofmann, M. (2021) *ACS Omega 6*, 6924–6933.^55^ Copyright 2021 American Chemical Society.

A set of alternative mechanisms proposed later by Habib et al.^55^ assume the binding of acetylene in the second ligand sphere of the W­(IV) ion, with one end of the molecule binding to the water ligand of W, while the other end interacts either directly or indirectly (via bridging water) with the carboxylic acid group of Asp13. In either case, this results in the polarization of the bound acetylene, enabling the addition of a OH^–^ to one of its C atoms to form a vinyl alcohol intermediate. Depending on the proposed mechanism, the hydroxy group of the alcohol descends either from the water ligand of W­(IV) (in case of direct interaction between acetylene and Asp13) or from the bridging water between acetylene and Asp13 (Figure 13B, C).^55^ Tautomerization of the vinyl alcohol to acetaldehyde occurs then as a spontaneous reaction, either in the first or second ligand shell of the active site. The barriers for initial acetylene hydration were calculated at around 63 kJ/mol for the pathway involving direct binding of Asp13 to the acetylene, and even 125 kJ/mol for the pathway involving bridging water. Conversely, the barrier for the proposed vinyl alcohol tautomerization has been predicted to depend on the size of the water cluster available in the active site, exhibiting lower values with increased numbers of water molecules.^55^

To date, there is not much consensus on the reaction mechanism of AH, and all proposed mechanisms have severe shortcomings. It is also puzzling that AH is so structurally similar to other Mo- or W-enzymes while being the only one not catalyzing a redox reaction. Therefore, we present a new mechanistic proposal for the enzyme, which combines a reductive and an oxidative partial reaction to the overall redox-neutral hydration of acetylene (Figure 14). The two partial reactions are inspired by the analogous reductive mechanism of benzoyl-CoA reductase and the oxidative one of AOR. We propose the reaction to be initiated by a PCET transfer from the W­(IV)-cofactor to the acetylene bound in the second ligand shell. The electron originates from the W­(IV) and may be transferred via the water ligand like in BamB, while the H^+^ may also come from the water ligand. This leads to a semireduced W­(V) center with a highly reactive bound ethylene radical. We suggest that this radical may react with the close-by side chain of Asp13, creating a new C–C bond with the carboxylic acid and initiating a rearrangement of the oxygen ligands, reminiscent of similar reactions known in radical-dependent mutases (e.g., glycerol dehydratases or methylmalonyl-CoA mutases).^56^ The rearranged adduct is then stabilized by transfer of the second e^–^ from W­(V) and another H^+^ from the W–OH ligand, yielding an oxidized W­(VI)-cofactor, while the acetylene/Asp13 adduct may either directly decompose into acetaldehyde and the semialdehyde form of Asp13 or form an acyloine of a covalently attached 1-hydroxyethyl ligand at the carbonyl group of Asp13. Since acyloines are chemically unstable, this intermediate would be cleaved to produce acetaldehyde and the Asp13-semialdehyde. Either of these processes may potentially be aided by acid–base chemistry from the close-by side chains of Trp179 or Trp472. With acetaldehyde already produced as a desired end product, the second half-reaction would then reoxidize the Asp13-semialdehyde in the active center, using the same mechanism as discussed for the AORs and ending back at the fully reduced W­(IV)-cofactor. The close proximity of Asp13 to the W–O ligand (below 3Å) indicates that H^–^ could indeed be directly transferred to the cofactor. Moreover, this mechanism would be in line with the requirement of a fully reduced enzyme to afford the reducing power needed for initial acetylene reduction, and all the following steps may be expected to proceed rapidly, diminishing the chances of trapping intermediates.

**Figure 14:**
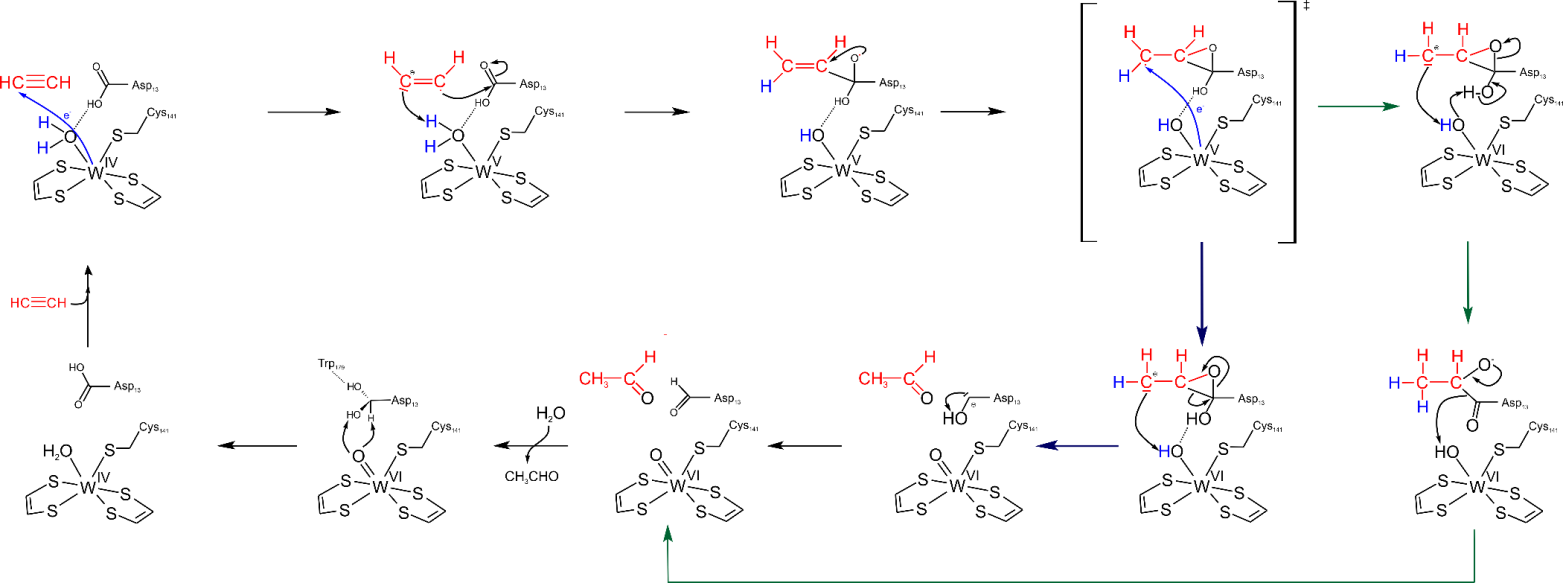
Proposed new mechanism for acetylene hydration. The reductive half-reaction is initiated by PCET from W­(IV) to acetylene, which then forms a covalent bond with Asp13 and is cleaved to acetaldehyde and Asp13-semialdehyde after the second PCET from W­(V). This cleavage may either occur directly (black arrows) or after forming an acyloine intermediate (green arrows). The second half-reaction then consists of reoxidation of Asp13-semialdehyde by the W­(VI)-cofactor using the same mechanism as in AOR.

## Functional Inorganic Models

Many attempts have been made to synthesize small metal–organic complexes as structural mimics or catalytically active replacements of the molybdenum or tungsten cofactors (reviewed in^57^). This work started in the late 1980s when the first structures of Mo- and W-enzymes and the variable metal/metallopterin compositions in the different enzyme families became known. Especially the laboratories of Holm and Garner provided early *mono*-and *bis*-dithiolene models of both Mo- and W-cofactors, either using more chemically stable carriers for the dithiolene moiety than the pyranopterin ring system present in the enzymes or trying to approximate the naturally occurring dithiolenes in model complexes. Consequently, the resulting models mimicked various aspects of molybdo- or tungstopterins, but did not allow to reproduce all effects of Mo- or W-cofactors sufficiently, or sometimes only allowed the insertion of other metals instead of Mo or W (e.g. refs 58, 59 ). However, a number of functional models were reported in recent years, which achieved reactivity with dithiolene ligation of the metal, albeit at low rates.^60^ Additional models ligating Mo or W not via dithiolene groups, but via N, O, or P ligands in the organic compounds, were constructed and often show higher catalytic efficiency than the “more natural” dithiolene-based models.^60^ They were also instrumental (while limited by their artificial nature) in correlating their known geometry to that of the active sites of the enzymes by similarities in their spectroscopic or electrochemical properties.^57^ A limited number of models have been reported to actually mimic the catalytic cycle and provide insight into proposed reaction intermediates, typically Mo-complexes with nonsulfur ligands.^61^ However, few chemical models have been prepared with W, mostly to compare them with the corresponding Mo-complexes. Interesting observations were that complexes containing W showed a more temperature-sensitive response to redox potential changes^57^ or a slower reactivity in oxo-transfer from the oxidized complex to organophosphine acceptors than the corresponding Mo-complexes.^60^ Targeted approaches to create model W-complexes were mostly attempted for reactions believed to be dependent or strongly enhanced by the presence of W, such as W-hydrogenase and acetylene hydratase models, which are presented as follows.

### W-co Model as Hydrogenase

The first reports of hydrogenase activity exhibited by tungsten dithiolene complexes are due to inorganic models of Mo/W proteins. In their pursuing a functional inorganic model of formate dehydrogenase, an unexpected evolution of H_2_ was reported to be coupled to the oxidation of a W­(IV)­(O)­[(S_2_C_2_Ph_2_)_2_]^−^ cluster to the W­(V) species.^62^

The possibility of the W-co acting as a hydrogenase-like catalyst was independently demonstrated in studies using *bis*(dithiolene)tungsten complexes, which mimic FOR (and AOR) active sites.^63^ The authors were able to catalyze the electroreduction of protons into hydrogen in acetic acid with a high rate constant of 100 s^–1^ and high overpotential (700 mV). Based on the experiments and DFT calculations, the authors proposed a mechanism that assumes the W­(IV)­O­(L^COOMe^)_2_ complex to be the active catalytic form responsible for proton reduction (Figure 15). In the first step, the proton binds to the W=O ligand and this is accompanied by 1e^–^ reduction which leads to a transient W­(III)–OH complex. In the next step, the transfer of a second H^+^and a coupled electron (either sequentially or in a coupled process) leads to the formation of a metal-bound hydride W­(IV)­H–OH complex. The formation of the H_2_ then proceeds through a transition state where the hydride atom recombines with the H^+^, forming the hydrogen as a product. The calculated ΔG barrier for such H_2_ evolution was 74.5 kJ mol^–1^ and the reaction had a significantly exergonic character (ΔG_r_ −116 kJ mol^–1^) which indicates that the reverse process, i.e. reduction of the model complex with H_2_, would require overcoming a very high barrier of 190 kJ mol^–1^.

**Figure 15:**
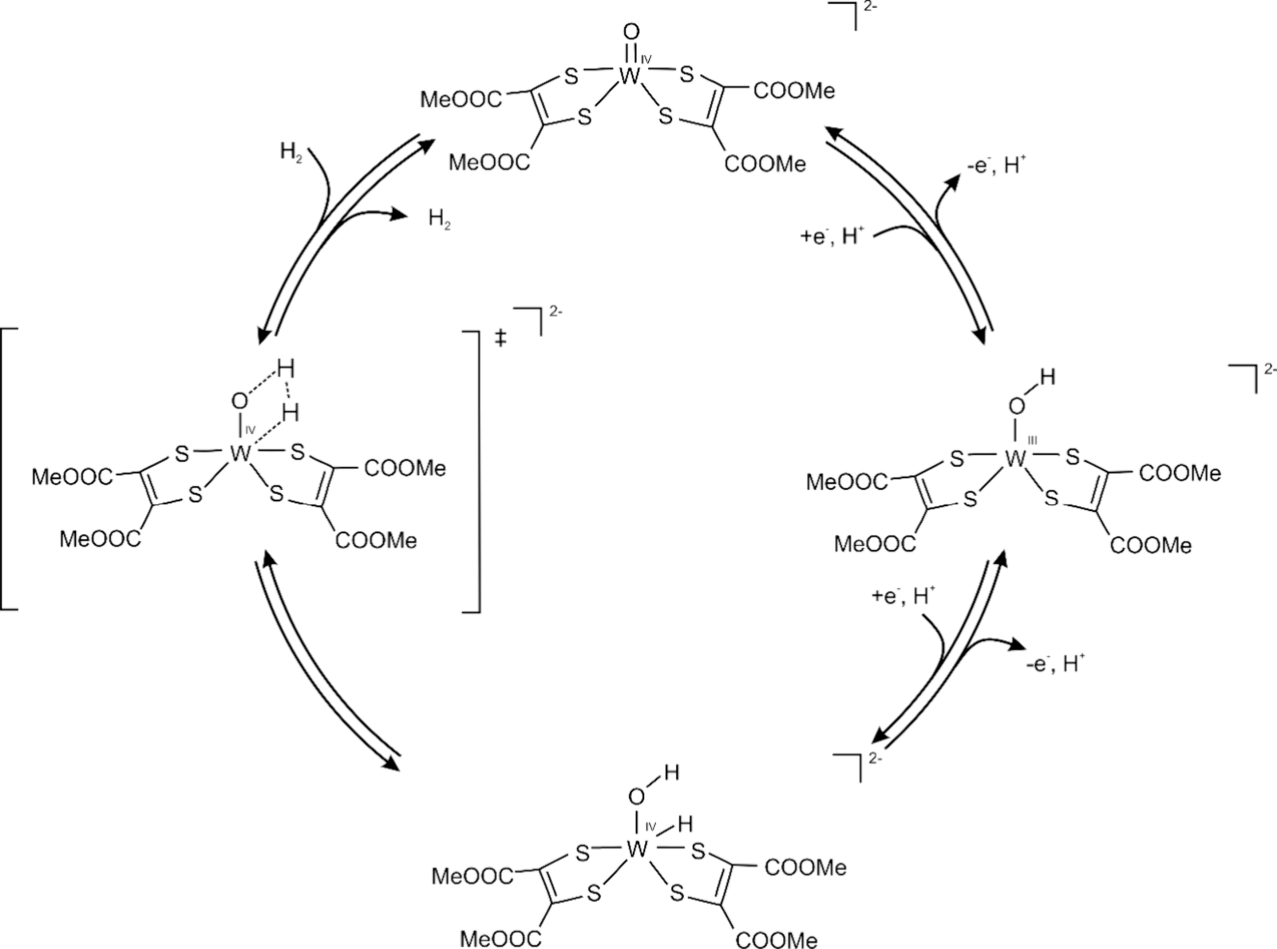
Mechanism explaining hydrogenase activity of W-co model complex and AOR. Figure adapted from the literature.^63^ Reproduced from Gomez-Mingot, M., Porcher, J. P., Todorova, T. K., Fogeron, T., Mellot-Draznieks, C., Li, Y., and Fontecave, M. (2015), *J. Phys. Chem. B 119*, 13524–13533.^63^ Copyright 2015 American Chemical Society.

### W-co Models for Acetylene Hydration

Since AH represents an enzyme specifically dependent on tungsten, many attempts have been made to emulate its chemistry by model compounds. Initial attempts yielded acetylene-binding W­(IV)-complexes, which did not catalyze its hydration,^64^ while a plethora of further acetylene-binding W-complexes has been synthesized in recent years, which ligate W via two different atoms per ligand (e.g., N/S or N/Se), rather than the two sulfurs of the naturally occurring dithiolenes.^65,66^ Many of these complexes are able to bind acetylene in their first ligation sphere, mimicking the initially proposed AH mechanisms. However, reactions with the bound acetylene have only been observed with unphysiological “hard” nucleophiles such as organophosphines,^67^ while “the direct nucleophilic attack of W-coordinated acetylene by water, as some computational studies of acetylene hydratase propose, is unlikely to occur”.^68^ In addition, many of the reported W-complexes or their Mo-analogues catalyze different reactions from those known from the respective enzymes, such as alkene epoxidation or phosphine hydroxylation.^64,69,70^ These shortcomings of recreating the reaction of AH with model complexes are also indicating that the current first-shell mechanistic proposals may not accurately represent the real reaction mechanism.

## Application of Tungsten Enzymes

### Biofuel Production Setup by Converting Carboxylic Acids to Alcohols (In Whole Cells and in Cell-Free System)

One of the first reported biotechnological applications involving W enzymes was on bioreduction of organic acids or aldehydes to the corresponding alcohols in batch cultures of *P. furiosus* grown jointly on starch and the organic acids or aldehydes intended to be turned over at 90 °C.^71^ The process yielded high rates of alcohols from various aliphatic and alkylaromatic acids (up to >60% conversion), while some aldehydes were disproportioned to acids and alcohols. The processes depended on joint activities of indigenous AOR and alcohol dehydrogenases (ADH) already present in the organism and did not extend to converting the main fermentation product acetate to ethanol. It was observed that the presence of some H_2_ in the atmosphere was necessary for the reduction of acids, although high concentrations inhibited the growth of the cells. Furthermore, the presence of acids in the fermentation media inhibited the growth of *P. furiosus*, which seemed to limit the biotechnological application. The process was further elucidated by Ni et al.^72^ who demonstrated efficient bioreduction of a broad range of acids to alcohols at a significantly lower temperature (40 °C) at pH 6.5 and at 5 bar of H_2_. Under these conditions, 10 mM loads of the substrates were transformed into the corresponding alcohols within 48h with yields of 20 to >99%. The authors speculate that the process is catalyzed by AOR and various ADHs together with membrane-bound and soluble hydrogenases utilizing H_2_ for the reduction of Fd and NAD­(P)^+^. Fermentative production of bioalcohol by *P. furiosus* was further developed to include ethanol,^73^ using a genetically modified strain of *P. furiosus* carrying a gene for an alcohol dehydrogenase (AdhA) from the thermophilic bacterium *Thermoanaerobacter* strain X514. This created a synthetic fermentation pathway at high temperatures (70 °C) for converting glucose to ethanol and CO_2_, with AOR and AdhA reducing the standard end product acetate to ethanol via acetaldehyde. Due to the wide substrate specificity of AOR, other carboxylic acids are converted to the corresponding alcohols as well. For example, with 40 mM butyrate added, 25 mM of butanol was produced along with 12 mM ethanol and 15 mM acetate.^73^ The reduction of acids to alcohols is driven by electrons from the oxidation of glucose, pyruvate or H_2_ in these cells.

After further modifying the AdhA-containing strain of *P. furiosus* by introducing the full operon encoding the carbon monoxide dehydrogenase/hydrogenase complex (CODH) from *Thermococcus onnurineus*, CO or H_2_ became accessible as electron donors for alcohol production. The obtained strain uses CO or H_2_ as sole sources of electrons to be funneled to Fds and NADP^+^ and utilized by AOR and AdhA in the reduction of acids to alcohols. The proof-of-concept process demonstrated the production of 70 mM isobutanol from 110 mM isobutyrate in a 3-day fermentation process. Interestingly, similar activities of *P. furiousus* (i.e., CO driven reduction) were also reported for the wild-type strain (DSM 3638) where cinnamic acid was reduced either by H_2_ or CO with comparable efficiency i.e., of approximately 70–80% of 10 mM substrate was converted at 40 °C.^74^

A similar report was recently published by Lemaire et al. for the acetogenic bacterium *C. autoethanogenum*, a strict anaerobe that uses CO as a sole source of carbon and electrons and produces AOR_*Ca*_.^27^ Some of the acetate produced during fermentation is further reduced to ethanol if CO is used as an electron donor. This explains the observed production of ethanol from H_2_/CO_2_/CO gas mixtures such as syngas, a prevalent industrial waste product, and allows to convert it to higher value-added products. Further attempts are ongoing to convert acetate to butyrate and caproate by optimizing the ethanol/acetate fermentation pathway of *Clostridium kluyveri* and subsequently convert these acids to alcohols via coupled AOR/ADH reactions, as well as Mo- or W-containing formate dehydrogenases involved in acetogenic CO_2_ fixation.^75–78^ The expected higher alcohols like butanol or hexanol from such a process represent much better potential biofuels or starting compounds for chemical synthesis.

In addition to the mentioned ongoing whole-cell biotransformations systems, a cell-free system was reported that uses recombinant AOR_*Aa*_ combined with a benzyl alcohol dehydrogenase to reduce benzoate via benzaldehyde to benzyl alcohol.^22^ AOR_*Aa*_ was shown to exhibit an alternative activity as hydrogenase and to use H_2_ as a reductant for the reduction of benzoate to benzaldehyde and NAD^+^ to NADH. The NADH produced by AOR_*Aa*_ is then driving the further reduction of benzaldehyde to benzyl alcohol by the added benzyl alcohol dehydrogenase. The process works at 30 °C and slightly acidic pH (6.5) with only 2.5% of H_2_ in the atmosphere, enabling the production of 0.1 mM benzyl alcohol in 30 min. The same system was also tested when whole cells of *A. evansii* containing overproduced AOR were mixed with isolated BaDH enzyme and NADH, yielding 0.3 mM benzyl alcohol in 4 h.^24^

### NADH Recycling System

The hydrogenase activity of AOR_*Aa*_ has been also used for NADH recycling purposes. The enzyme was added to a buffer containing 2 mM acetophenone, NAD^+^ and (*R*)-1-phenylethanol dehydrogenase with 2% of H_2_. The reaction proceeded at a constant rate for 2 days without slowing down, yielding 0.43 mM (*R*)-1-phenylethanol. The same reaction was tested also in a syngas atmosphere (59% N_2_, 40% CO, and 1% H_2_) without any deterioration of the enzyme activity.^22^

### AAA Cycle

An even more exciting application of AOR_*Aa*_ in a cell-free system was recently demonstrated for producing ATP from electricity.^25^ In this seminal work, a cell-free artificial ATP-producing enzymatic cycle was assembled in which propionate was first reduced by AOR to propionaldehyde in an electrochemical cell with hexamethyl viologen as a mediator of low redox potential. The propionaldehyde was then reoxidized by a CoA-acylating NADP^+^ dependent propionaldehyde dehydrogenase from *Rhodopseudomonas palustris* BisB18, and the produced propionyl-CoA was converted to propionyl-phosphate by phosphotransacetylase from Escherichia coli. Finally, propionyl-phosphate was converted back to the starting compound propionate by propionate kinase from E. coli, coupled to synthesizing ATP from ADP. The system was additionally supported by an NADP^+^ recycling system, using lactate dehydrogenase to restore NADP^+^. The system enables electricity-driven regeneration of ATP at the rate of 2.7 mmol cm^–2^ h^–1^ and faradaic efficiencies of up to 47% at an electrode potential of −0.6 V vs SHE.

### Fermentative Formation of Cyclohexane Derivatives

An interesting further biotechnological application of W-enzyme-dependent reactions would be the fermentative production of cyclohexane carboxylate or even cyclohexylmethanol. This process may be built on the ability of some syntrophic anaerobic bacteria such as *Syntrophus aciditrophicus* to ferment crotonate or benzoate to acetate and cyclohexane carboxylate, respectively.^79,80^ To achieve this, the pathway is split into an oxidative and a reductive branch, yielding acetate and cyclohexane carboxylate as the respective end products. Energy is conserved during oxidation of the substrates to acetate, particularly by converting acetyl-phosphate to acetate via acetate kinase, while the generated redox equivalents are recycled by reverting the degradation pathways of crotonyl-CoA to benzoyl-CoA and further to cyclohexane carbonyl-CoA. The latter part of the reductive pathway involves a W-dependent benzoyl-CoA reductase, which is required for reducing benzoyl-CoA to cyclohexa-1,5-diene-1-carboxyl-CoA as outlined above for the enzyme from *G. metallireducens*. The syntrophic nature of the organisms should allow the process to run in mixed cultures with other organisms feeding in,^81^ opening possible connections to create value-added products from waste disposal facilities degrading aliphatic or aromatic compounds or even gases (e.g., syngas). The produced acids (especially cyclohexane carboxylate) may even be further converted to the corresponding alcohols, which may be useful as biofuels or fine chemicals. This would require the presence of tungsten-containing AOR and appropriate alcohol dehydrogenases in the syntrophic strains, which may either be accomplished by artificially inserting genes for these enzymes in known bacterial strains or by screening for naturally occurring microbes exhibiting these traits.

## Conclusions

The biochemistry of tungstoenzymes has not been explored to the same level as for molybdoenzymes, mostly due to their high sensitivity to oxygen, difficulty in cultivating the respective microorganisms or lack of efficient recombinant expression platforms. Nevertheless, their unique chemical characteristics render them highly interesting for biotechnological applications, including sustainability or net zero emission efforts. Potential tungstoenzyme applications apply to the production of biofuels from waste streams, fermentative production of added value chemicals, H_2_-based technologies, as well as CO_2_ sequestration and utilization.

The nonstandard features of W-co introduced by the heaviest element used in biology opens also a new mechanistic opportunity for chemical catalysis, such as direct reduction of the benzene ring by BCR or acetylene hydration by AH. In this review, we propose that the catalytic mechanism of action of all obligately tungsten-dependent enzymes may proceed according to a common second-shell mechanism. Within the AOR family, the mechanistic analogies between the reductive mechanism of BCR and the (usually analyzed) oxidative one of AOR only become evident with the newly proposed second-shell mechanism, which also allows to be reverted for acid reduction. It is even more surprising, that the same structural characteristics of localizing W-co and a Fe_4_S_4_ cluster with some surrounding acid/base catalysts are not limited to the related enzymes of the AOR/WOR family, but have apparently extended to acetylene hydratase as otherwise unrelated DMSOR family member. We propose that this may be a result of convergent evolution which leads to a similar solution for the reaction mechanism.

It should be also underlined that several of the mechanisms proposed in this review are hypothetical, although based on structural, spectroscopic, and computational premises. They should be treated therefore as an open challenge to the community to be tested, confirmed, or falsified.

## Supplementary Material



## Notes

Table S1 with summary on obligate W-enzymes; Technical information on construction of the phylogenetic tree; List of the sequences selected for phylogenetic tree (PDF)
