# Supplementary material for: Obligately Tungsten-Dependent Enzymes—Catalytic Mechanisms, Models and Applications
Source: Biochemistry. 2025 May 5;64(10):2154–72. doi: 10.1021/acs.biochem.5c00116 (PMC12096430; doi:10.1021/acs.biochem.5c00116)
Supplement: Supplementary file 1 [file bi5c00116_si_001.pdf]

Supplementary information

Obligately tungsten-dependent enzymes – catalytic mechanisms, models and applications

Maciej Szaleniec[1]*, Johann Heider[2,3#]

1 - Jerzy Haber Institute of Catalysis and Surface Chemistry Polish Academy of Sciences

2 - Faculty of Biology, Philipps-Universität Marburg, Marburg, Germany

3 - Center for Synthetic Microbiology, Philipps-Universität Marburg, Marburg, Germany

Email: *maciej.szaleniec@ikifp.edu.pl; #heider@staff.uni-marburg.de

Table S1. Summary of the obligate W-enzymes. BV- benzyl viologen, MV – methyl viologen, HMV – hexamethyl viologen, TMV – tetramethyl viologen, DCPIP – dichloroindophenol, MB – methylene blue

Name of the enzyme	Subunit composition	Substrates	Activities	Electron carriers (artificial)	Cofactors	Structure PDB	Refs and notes
Aldehyde oxidoreductases from *Pyrococcus furiosus* (AOR_{Pf}) and other *Pyrococcus* and *Thermococcus* sp.	α_2 w/Fe^{2+}	aldehydes carboxylic acids	oxidation of aldehydes reduction of acids	ferredoxin (BV)	2x(W-bis-MPT-Mg, Fe_4S_4)	1AOR	[1-7] Oxygen sensitive
Aldehyde oxidoreductase from *Clostridium autoethanogenes* (AOR_{Ca})	α	aldehydes (unbranched and not hydrophilic) aliphatic acids (acetate)	oxidation of aldehydes reduction of acids	ferredoxin (MV, BV)	W-bis-MPT-Mg, Fe_4S_4	9G7J	[8]
Aldehyde oxidoreductase from *Desulfovibrio gigas* (AOR_{Dg})	α_2	aldehydes	oxidation of aldehydes	(BV, MV)	W-co Fe_4S_4		[9] Oxygen sensitive
Aldehyde oxidoreductase from *Moorella thermoacetica* (AOR_{Mt})	$\alpha\beta\gamma$?	aliphatic and aromatic aldehydes	oxidation of aldehydes	(MV, TMV)			[10]

		acids	Reduction of acids				
Aldehyde oxidoreductase<br>*Aromatoleum aromaticum* (AOR$_{Aa}$)<br>And other Betaproteobacteria	$(\alpha\beta)_n\gamma$	Wide spectrum<br>aldehydes<br>carboxylic acids<br>H_2	oxidation of aldehydes<br>oxidation of H_2<br>reduction of acids	NAD^+<br>(BV, MV, TMV, HMV, DCPIP, MB)	α: W-bis-MPT-Mg, Fe_4S_4<br>β: 4x Fe_4S_4<br>γ: FAD	8C0Z	[11-17]<br>O_2 tolerant
Tungsten oxidoreductase from *Eubacterium limosum* (WOR1$_{El}$)<br>or *Acetomicrobium mobile* (WOR1$_{Am}$)	αβγδε	aliphatic and aromatic aldehydes	oxidation of aldehydes	NAD^+<br>ferredoxin<br>(BV)	α: W-co, Fe_4S_4<br>β: 4x Fe_4S_4<br>γ: Fe_2S_2<br>δ: Fe_2S_2<br>2x Fe_4S_4<br>ε: 4x Fe_4S_4<br>Fe_2S_2<br>FMN		[18, 19]
Tungsten oxidoreductase from<br>*Acetomicrobium mobile* (WOR2$_{Am}$)	αβ?	aliphatic and aromatic aldehydes	oxidation of aldehydes	ferredoxin<br>(BV)	α: W-co, Fe_4S_4<br>β: 4x Fe_4S_4		[18, 19]
AOR from *B. massiliensis (AOR$_{Bm}$)*	α	Wide spectrum of aldehydes (aliphatic, aromatic, heterocyclic)		ferredoxin<br>(BV)	W-co<br>Fe_4S_4		[20]<br>O_2 insensitive

Glyceraldehyde-3-phosphate ferredoxin oxidoreductases from *Archaea* (GAPOR)	α	D-glyceraldehyde-3-phosphate	oxidation to 3-phosphoglycerate	ferredoxin (MV, BV)	W-bis-MPT-Mg, Fe_4S_4;		[7, 21-25] Potential Mo-co enzyme (*M. maripaludis*)
Glyceraldehyde-3-phosphate ferredoxin oxidoreductases from *Bacteria* (GOR)	$\alpha_2\beta_2$	glyceraldehyde-3-phosphate	oxidation to 3-phosphoglycerate	ferredoxin (MV, BV)	2x(α: W-bis-MPT-Mg, Fe_4S_4; β 4x Fe_4S_4)		[26]
Formaldehyde oxidoreductases (FOR) from *Archaea*	α_4	short-chain (C1-C4) aliphatic aldehydes glutaric semialdehyde	oxidation of aldehydes	ferredoxin (MV, BV)	4x(W-bis-MPT-Mg, Fe_4S_4)	1B25, 1B4N	[7, 27, 28]
W-dependent oxidoreductase from *P. furiosus* (WOR4)		unknown			W-co Fe_3S_4	6X6U 6X1O	[29, 30]
Aliphatic sulfonate ferredoxin oxidoreductase from *P. furiosus* (ASOR, formerly WOR5)	$\alpha_2\beta_2$	taurine aliphatic and aromatic aldehydes	Sulfonate elimination oxidation of aldehydes				

Class II benzoyl-CoA reductases (BCR)	$\alpha_2\beta_2$ module in megadalton complex	Benzoyl-CoA	aromatic ring reduction to dienoyl-CoA	NAD^+ ferredoxin MK	BamB: W-bis-MPT-Mg, Fe_4S_4<br>BamC: 3x Fe_4S_4<br>BamF Fe_2S_2<br>BamE 2x FAD 6x Fe_4S_4<br>BamD 2x Fe_4S_4 2x non-cubane Fe_4S_4<br>BamG Fe_2S_2<br>BamI 2x Fe_4S_4 Fe_2S_2<br>BamH 3x Fe_4S_4 Fe_2S_2<br>FAD	4Z3X<br>4Z3Y<br>4Z3Z,<br>4Z40<br>4Z3W	[31-35]
acetylene hydratase from *Syntrophotalea* (AH)<br>[DMSOR family]	α	acetylene	Hydratation to acetaldehyde	none	W-bis-MGD<br>Fe_4S_4	2E7Z	[36-39]

Sequence list used for phylogenetic analysis

The sequences were manually selected sequences from different clades to ensure that the sequence variability is well represented while the redundant entries are pruned. Then they were aligned by Clustal omega[40], and the tree was constructed using iTOL[41].

>A_carboniph WP_184307275.1 aldehyde ferredoxin oxidoreductase family protein [Anaerosolibacter carboniphilus]
>Accumulibacter_phosphatis_aor wp_034952824
>AccumulibacterWP_012807114.1 aldehyde ferredoxin oxidoreductase family protein [Accumulibacter sp.]
>Acetohalobium1 WP_013278442.1 aldehyde ferredoxin oxidoreductase family protein [Acetohalobium arabaticum]
>AcetohalobiumWP WP_013277952.1 aldehyde ferredoxin oxidoreductase family protein [Acetohalobium arabaticum]
>AcetohalobiumWP_013277925.1 aldehyde ferredoxin oxidoreductase family protein [Acetohalobium arabaticum]
>AcetohalobiumWP_013278442.1 aldehyde ferredoxin oxidoreductase family protein [Acetohalobium arabaticum]
>AcetohalobiumWP_013278469.1 aldehyde ferredoxin oxidoreductase family protein [Acetohalobium arabaticum]
>Acetomicrobium_h1 WP_057940722.1 aldehyde ferredoxin oxidoreductase family protein [Acetomicrobium hydrogeniformans]
>AcetomicrobiumWP_014805989.1 aldehyde ferredoxin oxidoreductase family protein [Acetomicrobium flavidum]
>AcetomicrobiumWP_014806007.1 aldehyde ferredoxin oxidoreductase family protein [Acetomicrobium mobile]
>AcetomicrobiumWP_014807295.1 aldehyde ferredoxin oxidoreductase family protein [Acetomicrobium flavidum]
>AcetomicrobiumWP_014807400.1 aldehyde ferredoxin oxidoreductase family protein [Acetomicrobium mobile]
>AcetomicrobiumWP_014807771.1 aldehyde ferredoxin oxidoreductase family protein [Acetomicrobium mobile]
>AcidilobusWP_013266680.1 aldehyde ferredoxin oxidoreductase family protein [Acidilobus saccharovorans]
>AcidilobusWP_013267166.1 aldehyde ferredoxin oxidoreductase family protein [Acidilobus saccharovorans]
>AcidilobusWP_013267212.1 aldehyde ferredoxin oxidoreductase family protein [Acidilobus saccharovorans]
>Acidovorax_sp.JS42_aor Abm44050
>AciduliprofundumWP_008082735.1 aldehyde ferredoxin oxidoreductase family protein [Candidatus Aciduliprofundum boonei]
>AciduliprofundumWP_008083804.1 aldehyde ferredoxin oxidoreductase family protein [Candidatus Aciduliprofundum boonei]
>AciduliprofundumWP_012997482.1 aldehyde ferredoxin oxidoreductase family protein [Candidatus Aciduliprofundum boonei]
>AdlercreutziaWP_022739251.1 MULTISPECIES: aldehyde ferredoxin oxidoreductase [Adlercreutzia]
>Aerophobetes1 MEE9191837.1 aldehyde ferredoxin oxidoreductase family protein [Candidatus Aerophobetes bacterium]
>Af1 SIN67281.1 aldehyde:ferredoxin oxidoreductase [Acetomicrobium flavidum]
>Af10 NLG95647.1 aldehyde ferredoxin oxidoreductase family protein [Acetomicrobium flavidum]
>Af2 NLG95647.1 aldehyde ferredoxin oxidoreductase family protein [Acetomicrobium flavidum] = Af1
>Af3 NLG95692.1 aldehyde ferredoxin oxidoreductase family protein [Acetomicrobium flavidum]
>Af4 SIN69259.1 aldehyde:ferredoxin oxidoreductase [Acetomicrobium flavidum]=Af5
>Af5 NLG95230.1 aldehyde ferredoxin oxidoreductase family protein [Acetomicrobium flavidum] = Af4
>Af6 NLG95572.1 aldehyde ferredoxin oxidoreductase family protein [Acetomicrobium flavidum]
>Af7 NLG95505.1 aldehyde ferredoxin oxidoreductase family protein [Acetomicrobium flavidum]
>Af8 NLG95344.1 aldehyde:ferredoxin oxidoreductase [Acetomicrobium flavidum]
>Af9 SIN75019.1 aldehyde:ferredoxin oxidoreductase [Acetomicrobium flavidum]
>Ah2 HHZ04958.1 aldehyde ferredoxin oxidoreductase family protein [Acetomicrobium hydrogeniformans]
>Ah3 WP_057940733.1 aldehyde ferredoxin oxidoreductase family protein [Acetomicrobium hydrogeniformans]
>Ah4 WP_057940909.1 aldehyde ferredoxin oxidoreductase family protein [Acetomicrobium hydrogeniformans]
>Ah5 WP_040347812.1 aldehyde ferredoxin oxidoreductase [Acetomicrobium hydrogeniformans]
>AlkalilimnicolaWP_011629041.1 aldehyde ferredoxin oxidoreductase family protein [Alkalilimnicola ehrlichii]
>AlkaliphilusWP_012065602.1 aldehyde ferredoxin oxidoreductase family protein [Alkaliphilus metalliredigens]
>Am1 WP_014805989.1 aldehyde ferredoxin oxidoreductase family protein [Acetomicrobium mobile]
>Am2 WP_014807771.1 aldehyde ferredoxin oxidoreductase family protein [Acetomicrobium mobile]
>Am3 WP_014807400.1 aldehyde ferredoxin oxidoreductase family protein [Acetomicrobium mobile]
>Am4 WP_211205155.1 aldehyde ferredoxin oxidoreductase family protein [Acetomicrobium mobile]
>Am5 WP_014807295.1 aldehyde ferredoxin oxidoreductase [Acetomicrobium mobile]
>AminobacteriumWP_013049248.1 aldehyde ferredoxin oxidoreductase family protein [Aminobacterium colombiense]
>AnaerolineaWP_013559877.1 aldehyde ferredoxin oxidoreductase family protein [Anaerolinea thermophila]
>AnaerolineaWP_049784858.1 aldehyde ferredoxin oxidoreductase family protein [Anaerolinea thermophila]
>Anaeromicrobium_sed PAB59243.1 aldehyde:ferredoxin oxidoreductase [Anaeromicrobium sediminis]
>AOR_Carboxydothermus_hydrogenoformans wp_011343719
>AOR_Clostridium_formicaceticum wp_070964744
>AOR_Coprothermobacter_platensis WP_018963763
>AOR_Desulfovibrio_gigas wp_021759675
>AOR_Hippea_jasoniae wp_035589419
>AOR_Methanospirillum_hungatei_JF1 wp_011448034
>AOR_Moorella_thermoacetica wp_071520516
>AOR_Palaeococcus_ferrophilus wp_048150761
>AOR_Peptoclostridium_acidaminophilum ahm57984
>AOR_Pyrobaculum_aerophilum wp_011007373
>AOR_Pyrococcus_furiosus WP_011011461
>AOR_Sedimentibacter WP_145080825.1 aldehyde ferredoxin oxidoreductase [Sedimentibacter saalensis]
>AOR_Thermococcus_celericrescens wp_058938613
>AOR_Thermococcus_litoralis wp_004070105
>AOR_Thermococcus_paralvinellae_ES-1 wp_042681437

>AOR_Thermococcus_sibiricus wp_048160352
>AOR1_A_aromaticum WP_011238843.1 tungsten-containing aldehyde ferredoxin oxidoreductase (AOR-1) [Aromatoleum aromaticum]
>AOR1_A_tolulyticum WP_076601030.1 aldehyde ferredoxin oxidoreductase [Aromatoleum tolulyticum]
>AOR1_Acidiferrobacteraceae MBP49720.1 aldehyde ferredoxin oxidoreductase [Acidiferrobacteraceae bacterium]
>AOR1_Acidobacteria HCH37291.1 aldehyde ferredoxin oxidoreductase [Acidobacteria bacterium]
>AOR1_Amphritea WP_124927150.1 aldehyde ferredoxin oxidoreductase [Amphritea balenae]
>AOR1_Desulfovibrio WP_051261678.1 aldehyde ferredoxin oxidoreductase family protein [Desulfovibrio inopinatus]
>AOR1_Euryarchaeota TLZ59386.1 aldehyde ferredoxin oxidoreductase, partial [Euryarchaeota archaeon]
>AOR1_Myxococcales MSP15818.1 aldehyde ferredoxin oxidoreductase [Myxococcales bacterium]
>AOR1_S_selenatireducens PLX61918.1 aldehyde ferredoxin oxidoreductase [Sedimenticola selenatireducens]
>AOR1_S_thiotaurini TVT54171.1 aldehyde ferredoxin oxidoreductase [Sedimenticola thiotaurini]
>AOR1_Sulfuritalea WP_041098475.1 aldehyde ferredoxin oxidoreductase [Sulfuritalea hydrogenivorans]
>AOR1_Thauera WP_107220490.1 aldehyde ferredoxin oxidoreductase [Thauera aromatica]
>ArchaeoglobusWP_010877537.1 aldehyde ferredoxin oxidoreductase family protein [Archaeoglobus fulgidus]
>ArchaeoglobusWP_010877847.1 MULTISPECIES: aldehyde ferredoxin oxidoreductase family protein [Archaeoglobus]
>ArchaeoglobusWP_010879770.1 MULTISPECIES: aldehyde ferredoxin oxidoreductase family protein [Archaeoglobus]
>Aromatoleum_aromaticum_AOR CAI08971.1
>AromatoleumWP_011238652.1 aldehyde ferredoxin oxidoreductase family protein [Aromatoleum aromaticum]
>AromatoleumWP_011238843.1 aldehyde ferredoxin oxidoreductase C-terminal domain-containing protein [Aromatoleum aromaticum]
>Azoarcus_toluclasticus_AOR wp_228216202
>B_tuaregi WP_187143740.1 aldehyde ferredoxin oxidoreductase family protein [Bacillus tuaregi]
>Bacillota1 MCR4402872.1 aldehyde ferredoxin oxidoreductase family protein [Bacillota bacterium]
>BamB_BF ADJ94019.1 putative benzoate-degrading protein BamB, partial [Clostridia bacterium enrichment culture clone BF]
>BamB_Desulfococcus WP_020876803.1 hypothetical protein [Desulfococcus multivorans]
>BamB_Desulfosarcina_cetonica WP_054701953.1 aldehyde dehydrogenase [Desulfosarcina cetonica]
>BamB_Desulfosarcina_widdeli WP_155307761.1 aldehyde dehydrogenase [Desulfosarcina widdelii]
>BamB_Dethiosulfatarculus WP_044348233.1 aldehyde dehydrogenase [Dethiosulfatarculus sandiegensis]
>BamB_Geobacter_bemidjiensis WP_012529875.1 aldehyde dehydrogenase [Geobacter bemidjiensis]
>BamB_Rhodospirillaceae MAF50261.1 aldehyde dehydrogenase [Rhodospirillaceae bacterium]
>BamB_Syntrophorhabdus WP_028893812.1 aldehyde dehydrogenase [Syntrophorhabdus aromaticivorans]
>BamB-1_Geobacter_metallireducens wp_004514579
>BamB-2_Geobacter_metallireducens wp_004512044
>Bathyarchaeota_2 HGF75185.1 aldehyde ferredoxin oxidoreductase [Candidatus Bathyarchaeota archaeon]
>Bathyarchaeota2 UCH37534.1 MAG: aldehyde ferredoxin oxidoreductase family protein [Candidatus Bathyarchaeota archaeon]
>BipolaricaulisWP_122030599.1 aldehyde ferredoxin oxidoreductase family protein [Candidatus Bipolaricaulis anaerobius]
>BipolaricaulisWP_122031732.1 aldehyde ferredoxin oxidoreductase family protein [Candidatus Bipolaricaulis anaerobius]
>BrevefilumWP_087862161.1 aldehyde ferredoxin oxidoreductase family protein [Brevefilum fermentans]
>BrevefilumWP_087862902.1 aldehyde ferredoxin oxidoreductase family protein [Brevefilum fermentans]
>Brevibacillus WP_003387830.1 MULTISPECIES: aldehyde ferredoxin oxidoreductase family protein [Brevibacillus]
>C_aceticum WP_044825567.1 aldehyde ferredoxin oxidoreductase family protein [Clostridium aceticum]
>C_autoethanogenum AGY74327.1 aldehyde ferredoxin oxidoreductase family protein [Clostridium autoethanogenum DSM 10061]
>CaldilineaWP_014432542.1 MULTISPECIES: aldehyde ferredoxin oxidoreductase family protein [Caldilinea]
>Caldimonas_manganoxidans_AOR WP_019559976
>CaldisericumWP_014452563.1 aldehyde ferredoxin oxidoreductase family protein [Caldisericum exile]
>CaldisphaeraWP_015231943.1 aldehyde ferredoxin oxidoreductase family protein [Caldisphaera lagunensis]
>CaldisphaeraWP_015232470.1 aldehyde ferredoxin oxidoreductase family protein [Caldisphaera lagunensis]
>CaldisphaeraWP_015232762.1 aldehyde ferredoxin oxidoreductase family protein [Caldisphaera lagunensis]
>CaldivirgaWP_012185328.1 aldehyde ferredoxin oxidoreductase family protein [Caldivirga maquilingensis]
>CaldivirgaWP_012185404.1 aldehyde ferredoxin oxidoreductase family protein [Caldivirga maquilingensis]
>CaldivirgaWP_012185766.1 aldehyde ferredoxin oxidoreductase family protein [Caldivirga maquilingensis]
>CaldivirgaWP_012186183.1 aldehyde ferredoxin oxidoreductase family protein [Caldivirga maquilingensis]
>CarboxydocellaWP_078665510.1 MULTISPECIES: aldehyde ferredoxin oxidoreductase family protein [Carboxydocella]
>CarboxydocellaWP_107754068.1 aldehyde ferredoxin oxidoreductase family protein [Carboxydocella thermautotrophica]
>CarboxydothermusWP_011343719.1 aldehyde ferredoxin oxidoreductase family protein [Carboxydothermus hydrogenoformans]
>CarboxydothermusWP_011344239.1 aldehyde ferredoxin oxidoreductase family protein [Carboxydothermus hydrogenoformans]
>CarboxydothermusWP_011344607.1 aldehyde ferredoxin oxidoreductase family protein [Carboxydothermus hydrogenoformans]
>ChloroflexusWP_012616440.1 aldehyde ferredoxin oxidoreductase family protein [Chloroflexus aggregans]
>ChondromycesWP_063796372.1 aldehyde ferredoxin oxidoreductase family protein [Chondromyces crocatus]
>CloacibacillusWP_084002271.1 aldehyde ferredoxin oxidoreductase family protein [Cloacibacillus porcorum]
>CloacimonasWP_015424451.1 aldehyde ferredoxin oxidoreductase family protein [Candidatus Cloacimonas acidaminovorans]
>Clostridium_homoprop WP_052221717.1 aldehyde ferredoxin oxidoreductase family protein [Clostridium homopropionicum]
>ClostridiumNP_348637.1 aldehyde:ferredoxin oxidoreductase [Clostridium acetobutylicum ATCC 824]
>ClostridiumYP_001254438.1 aldehyde ferredoxin oxidoreductase [Clostridium botulinum A str. ATCC 3502]
>CoprothermobacterWP_012543863.1 aldehyde ferredoxin oxidoreductase family protein [Coprothermobacter proteolyticus]
>CoprothermobacterWP_012544159.1 aldehyde ferredoxin oxidoreductase family protein [Coprothermobacter proteolyticus]
>CoprothermobacterWP_012544409.1 aldehyde ferredoxin oxidoreductase family protein [Coprothermobacter proteolyticus]
>Crenarchaeota NOZ30770.1 aldehyde ferredoxin oxidoreductase family protein [Crenarchaeota archaeon]
>CryptobacteriumWP_012802626.1 aldehyde ferredoxin oxidoreductase [Cryptobacterium curtum]
>CycloclasticusWP_015006918.1 MULTISPECIES: aldehyde ferredoxin oxidoreductase family protein [Cycloclasticus]
>D_inopinatus WP_027183050.1 aldehyde ferredoxin oxidoreductase [Desulfovibrio inopinatus]
>DeferribacterWP_013007490.1 aldehyde ferredoxin oxidoreductase family protein [Deferribacter desulfuricans]
>DehalobacteriumWP_089609655.1 aldehyde ferredoxin oxidoreductase family protein [Dehalobacterium formicoaceticum]
>Deltaproteobacteria__1 MBW2610198.1 aldehyde ferredoxin oxidoreductase family protein [Deltaproteobacteria bacterium]
>DenitrobacteriumWP_066663943.1 aldehyde ferredoxin oxidoreductase [Denitrobacterium detoxificans]
>DesulfatibacillumWP_012609848.1 aldehyde ferredoxin oxidoreductase family protein [Desulfatibacillum aliphaticivorans]
>DesulfatibacillumWP_015949201.1 aldehyde ferredoxin oxidoreductase family protein [Desulfatibacillum aliphaticivorans]

>DesulfatibacillumWP_015949411.1 aldehyde ferredoxin oxidoreductase family protein [Desulfatibacillum aliphaticivorans]
>DesulfobaculaWP_014955658.1 aldehyde ferredoxin oxidoreductase family protein [Desulfobacula toluolica]
>DesulfobaculaWP_014955952.1 MULTISPECIES: aldehyde ferredoxin oxidoreductase family protein [Desulfobacula]
>DesulfobaculaWP_014956090.1 aldehyde ferredoxin oxidoreductase family protein [Desulfobacula toluolica]
>DesulfobaculaWP_014959515.1 aldehyde ferredoxin oxidoreductase family protein [Desulfobacula toluolica]
>DesulfobaculaWP_041279157.1 aldehyde ferredoxin oxidoreductase family protein [Desulfobacula toluolica]
>DesulfofundulusAEG13808.1 Aldehyde ferredoxin oxidoreductase [Desulfofundulus kuznetsovii DSM 6115]
>DesulfofundulusAEG14201.1 Aldehyde ferredoxin oxidoreductase [Desulfofundulus kuznetsovii DSM 6115]
>DesulfofundulusAEG14204.1 Aldehyde ferredoxin oxidoreductase [Desulfofundulus kuznetsovii DSM 6115]
>DesulfofundulusAEG14238.1 Aldehyde ferredoxin oxidoreductase [Desulfofundulus kuznetsovii DSM 6115]
>DesulfofundulusAEG16449.1 Aldehyde ferredoxin oxidoreductase [Desulfofundulus kuznetsovii DSM 6115]
>DesulfofundulusAEG16576.1 Aldehyde ferredoxin oxidoreductase [Desulfofundulus kuznetsovii DSM 6115]
>DesulfohalobiumWP_015752269.1 aldehyde ferredoxin oxidoreductase family protein [Desulfohalobium retbaense]
>DesulfohalobiumWP_015752737.1 aldehyde ferredoxin oxidoreductase family protein [Desulfohalobium retbaense]
>DesulfomicrobiumWP_015774185.1 aldehyde ferredoxin oxidoreductase family protein [Desulfomicrobium baculatum]
>DesulfomicrobiumWP_015774325.1 aldehyde ferredoxin oxidoreductase family protein [Desulfomicrobium baculatum]
>DesulfomonileWP_014809790.1 aldehyde ferredoxin oxidoreductase [Desulfomonile tiedjei]
>DesulforapulumWP_012662573.1 aldehyde ferredoxin oxidoreductase family protein [Desulforapulum autotrophicum]
>DesulforapulumWP_012662851.1 aldehyde ferredoxin oxidoreductase family protein [Desulforapulum autotrophicum]
>DesulforapulumWP_015903312.1 putrescine aminotransferase [Desulforapulum autotrophicum]
>DesulforapulumWP_015905666.1 aldehyde ferredoxin oxidoreductase family protein [Desulforapulum autotrophicum]
>DesulforapulumWP_015906192.1 aldehyde ferredoxin oxidoreductase family protein [Desulforapulum autotrophicum]
>DesulfoscipioWP_006523855.1 aldehyde ferredoxin oxidoreductase family protein [Desulfoscipio gibsoniae]
>DesulfosporosinusWP_014183768.1 aldehyde ferredoxin oxidoreductase family protein [Desulfosporosinus orientis]
>DesulfosporosinusWP_014184881.1 aldehyde ferredoxin oxidoreductase family protein [Desulfosporosinus orientis]
>DesulfosudisWP_012175191.1 aldehyde ferredoxin oxidoreductase family protein [Desulfosudis oleivorans]
>DesulfosudisWP_012175705.1 aldehyde ferredoxin oxidoreductase family protein [Desulfosudis oleivorans]
>DesulfurellaWP_025391237.1 aldehyde ferredoxin oxidoreductase family protein [Desulfurella acetivorans]
>DesulfurococcusWP_012608260.1 aldehyde ferredoxin oxidoreductase family protein [Desulfurococcus amylolyticus]
>DesulfurococcusWP_048058877.1 aldehyde ferredoxin oxidoreductase family protein [Desulfurococcus amylolyticus]
>Dethiobacter MBS3982577.1 aldehyde ferredoxin oxidoreductase family protein [Dethiobacter sp.]
>DL31_WP_014050780.1 aldehyde ferredoxin oxidoreductase family protein [halophilic archaeon DL31]
>DL31_WP_014051689.1 aldehyde ferredoxin oxidoreductase family protein [halophilic archaeon DL31]
>Dmultivorans WP_020877975.1 hypothetical protein [Desulfococcus multivorans]
>Dthioautotrophicum WP_045218114.1 aldehyde ferredoxin oxidoreductase [Desulfonatronum thioautotrophicum]
>Ecallanderi2 WP_217415695.1 aldehyde ferredoxin oxidoreductase family protein [Eubacterium callanderi]
>Ecallanderi3 WP_177190822.1 aldehyde ferredoxin oxidoreductase [Eubacterium callanderi]
>EggerthellaWP_009305283.1 MULTISPECIES: aldehyde ferredoxin oxidoreductase [Eggerthella]
>Elimosum2 WP_133968806.1 aldehyde ferredoxin oxidoreductase family protein [Eubacterium limosum]
>Elimosum3 WP_058693892.1 hypothetical protein [Eubacterium limosum]
>Emaltosivorans WP_074617211.1 hypothetical protein [Eubacterium maltosivorans]
>Eubacterium_callanderi WP_180493925.1 aldehyde ferredoxin oxidoreductase family protein [Eubacterium callanderi]
>Eubacterium_limosum WP_133966303.1 aldehyde ferredoxin oxidoreductase family protein [Eubacterium limosum]
>EubacteriumWP_013378673.1 aldehyde ferredoxin oxidoreductase family protein [Eubacterium callanderi]
>EubacteriumWP_013380059.1 MULTISPECIES: aldehyde ferredoxin oxidoreductase family protein [Eubacterium]
>F_globusWP_012965729.1 aldehyde ferredoxin oxidoreductase family protein [Ferroglobus placidus]
>F_globusWP_012966791.1 aldehyde ferredoxin oxidoreductase family protein [Ferroglobus placidus]
>Ferroglobus1 WP_290900222.1 aldehyde ferredoxin oxidoreductase N-terminal domain-containing protein, partial [Ferroglobus sp.]
>FervidicoccusAFH42187.1 aldehyde:ferredoxin oxidoreductase [Fervidicoccus fontis Kam940]
>FervidicoccusAFH42217.1 aldehyde:ferredoxin oxidoreductase [Fervidicoccus fontis Kam940]
>FervidicoccusAFH42594.1 Aldehyde:ferredoxin oxidoreductase [Fervidicoccus fontis Kam940]
>FervidicoccusAFH42897.1 formaldehyde:ferredoxin oxidoreductase [Fervidicoccus fontis Kam940]
>FfAOR PMB76015.1 aldehyde ferredoxin oxidoreductase [Fervidicoccus fontis]
>FlexistipesWP_013886533.1 aldehyde ferredoxin oxidoreductase family protein [Flexistipes sinusarabici]
>FOR_Pyrococcus_abyssi WP_010868317
>FOR_Pyrococcus_furiosus_DSM_3638 aal81327 (WP_014835355)
>FOR_Thermofilum_sp._ex4484_82 oyt29225
>GAPOR_Pyrococcus_abyssi wp_010868698
>GAPOR_Pyrococcus_furiosus wp_011011581
>GAPOR_Thermococcus_onnurineus wp_012572460
>GeoglobusWP_048091331.1 aldehyde ferredoxin oxidoreductase family protein [Geoglobus acetivorans]
>GeoglobusWP_048093536.1 aldehyde ferredoxin oxidoreductase family protein [Geoglobus acetivorans]
>GeosporobacterWP_069980992.1 aldehyde ferredoxin oxidoreductase family protein [Geosporobacter ferrireducens]
>GeosporobacterWP_083273293.1 aldehyde ferredoxin oxidoreductase family protein [Geosporobacter ferrireducens]
>GOR_Alkalibacter WP_073270162.1 aldehyde:ferredoxin oxidoreductase [Alkalibacter saccharofermentans]
>GOR_Caldicellulosiruptor_bescii WP_015907364
>GOR_Caloramator WP_103895832.1 aldehyde:ferredoxin oxidoreductase [Caloramator fervidus]
>GOR_Coprothermobacter WP_018963763.1 aldehyde ferredoxin oxidoreductase [Coprothermobacter platensis]
>GOR_Desulforudis WP_012301297.1 aldehyde ferredoxin oxidoreductase [Candidatus Desulforudis audaxviator]
>GOR_Desulfosporosinus WP_092332506.1 aldehyde:ferredoxin oxidoreductase [Desulfosporosinus hippei]
>GOR_Desulfuribacillus WP_069642500.1 aldehyde:ferredoxin oxidoreductase [Desulfuribacillus alkaliarsenatis]
>GOR_Heliorestis WP_153726138.1 aldehyde:ferredoxin oxidoreductase [Heliorestis convoluta]
>GOR_Methanosarcina WP_011022903.1 aldehyde ferredoxin oxidoreductase family protein [Methanosarcina acetivorans]
>GOR_Pseudobacteroides WP_036938755.1 aldehyde ferredoxin oxidoreductase [Pseudobacteroides cellulosolvens]
>GOR_Thermincola WP_052218011.1 aldehyde ferredoxin oxidoreductase family protein [Thermincola ferriacetica]
>GottschalkiaWP_014968633.1 aldehyde ferredoxin oxidoreductase family protein [Gottschalkia acidurici]

>GranulosicoccusWP_088915747.1 aldehyde ferredoxin oxidoreductase family protein [Granulosicoccus antarcticus]
>GudongellaWP_128425357.1 aldehyde ferredoxin oxidoreductase family protein [Gudongella oleilytica]
>GudongellaWP_128426560.1 aldehyde ferredoxin oxidoreductase family protein [Gudongella oleilytica]
>H_feraxWP_004041291.1 aldehyde ferredoxin oxidoreductase family protein [Haloferax volcanii]
>H_terrigenaWP_012945739.1 aldehyde ferredoxin oxidoreductase family protein [Haloterrigena turkmenica]
>H_terrigenaWP_012945759.1 aldehyde ferredoxin oxidoreductase family protein [Haloterrigena turkmenica]
>HalalkaliarchaeumWP_119820255.1 aldehyde ferredoxin oxidoreductase family protein [Halalkaliarchaeum desulfuricum]
>HalalkaliarchaeumWP_119821083.1 aldehyde ferredoxin oxidoreductase family protein [Halalkaliarchaeum desulfuricum]
>HalalkaliarchaeumWP_119821396.1 aldehyde ferredoxin oxidoreductase family protein [Halalkaliarchaeum desulfuricum]
>HalanaeroarchaeumWP_050048959.1 aldehyde ferredoxin oxidoreductase family protein [Halanaeroarchaeum sulfurireducens]
>HalanaeroarchaeumWP_050049090.1 aldehyde ferredoxin oxidoreductase family protein [Halanaeroarchaeum sulfurireducens]
>HalanaerobiumWP_013405269.1 aldehyde ferredoxin oxidoreductase family protein [Halanaerobium hydrogeniformans]
>HaliangiumWP_096058599.1 aldehyde ferredoxin oxidoreductase family protein [Haliangium ochraceum]
>HaloarculaWP_011223551.1 aldehyde ferredoxin oxidoreductase family protein [Haloarcula marismortui]
>HaloarculaWP_011224011.1 MULTISPECIES: aldehyde ferredoxin oxidoreductase family protein [Haloarcula]
>HalobiformaWP_076738677.1 aldehyde ferredoxin oxidoreductase family protein [Halobiforma lacisalsi]
>HalocellaWP_125991083.1 aldehyde ferredoxin oxidoreductase family protein [Halocella sp. SP3-1]
>HalodesulfurarchaeumWP_070365771.1 aldehyde ferredoxin oxidoreductase family protein [Halodesulfurarchaeum formicicum]
>HalodesulfurarchaeumWP_083258919.1 aldehyde ferredoxin oxidoreductase C-terminal domain-containing protein [Halodesulfurarchaeum formicicum]
>HalogeometricumWP_006055210.1 aldehyde ferredoxin oxidoreductase family protein [Halogeometricum borinquense]
>HalolaminaWP_014052500.1 aldehyde ferredoxin oxidoreductase family protein [Halolamina sp.]
>HalopenitusWP_096390100.1 aldehyde ferredoxin oxidoreductase family protein [Halopenitus persicus]
>HalopigerWP_013881236.1 aldehyde ferredoxin oxidoreductase family protein [Halopiger xanaduensis]
>HaloplanusWP_121919846.1 aldehyde ferredoxin oxidoreductase family protein [Haloplanus aerogenes]
>HalorubrumWP_015910101.1 aldehyde ferredoxin oxidoreductase family protein [Halorubrum lacusprofundi]
>HalorubrumWP_015910194.1 aldehyde ferredoxin oxidoreductase family protein [Halorubrum lacusprofundi]
>HalostagnicolaWP_049954362.1 aldehyde ferredoxin oxidoreductase family protein [Halostagnicola larsenii]
>HaloterrigenaWP_012943522.1 aldehyde ferredoxin oxidoreductase family protein [Haloterrigena turkmenica]
>HeliomicrobiumWP_012282098.1 aldehyde ferredoxin oxidoreductase family protein [Heliomicrobium modesticaldum]
>HerpetosiphonABX06226.1 Aldehyde ferredoxin oxidoreductase [Herpetosiphon aurantiacus DSM 785]
>HippeaWP_013681800.1 aldehyde ferredoxin oxidoreductase family protein [Hippea maritima]
>HippeaWP_013681845.1 aldehyde ferredoxin oxidoreductase family protein [Hippea maritima]
>HydrogenophilusWP_119335446.1 aldehyde ferredoxin oxidoreductase family protein [Hydrogenophilus thermoluteolus]
>HyperthermusWP_011821541.1 aldehyde ferredoxin oxidoreductase family protein [Hyperthermus butylicus]
>HyperthermusWP_110138747.1 aldehyde ferredoxin oxidoreductase family protein [Hyperthermus butylicus]
>IAA_Ferroglobus WP_012966791.1 aldehyde ferredoxin oxidoreductase family protein [Ferroglobus placidus]
>Ignicoccus WP_012123175.1 aldehyde ferredoxin oxidoreductase family protein [Ignicoccus hospitalis]
>Infirmifilum WP_052884847.1 aldehyde ferredoxin oxidoreductase family protein [Infirmifilum uzonense]
>IsosphaeraWP_013565788.1 aldehyde ferredoxin oxidoreductase family protein [Isosphaera pallida]
>KorarchaeumWP_012308670.1 aldehyde ferredoxin oxidoreductase family protein [Candidatus Korarchaeum cryptofilum]
>KorarchaeumWP_012308930.1 aldehyde ferredoxin oxidoreductase family protein [Candidatus Korarchaeum cryptofilum]
>KorarchaeumWP_012309423.1 aldehyde ferredoxin oxidoreductase family protein [Candidatus Korarchaeum cryptofilum]
>KorarchaeumWP_083758138.1 aldehyde ferredoxin oxidoreductase family protein [Candidatus Korarchaeum cryptofilum]
>KribbellaWP_012919754.1 aldehyde ferredoxin oxidoreductase family protein [Kribbella flavida]
>MarinitogaWP_014296623.1 MULTISPECIES: aldehyde ferredoxin oxidoreductase family protein [Marinitoga]
>MethanocellaWP_012034620.1 aldehyde ferredoxin oxidoreductase family protein [Methanocella arvoryzae]
>MethanocellaWP_012035347.1 aldehyde ferredoxin oxidoreductase family protein [Methanocella arvoryzae]
>MethanocorpusculumWP_011832564.1 aldehyde ferredoxin oxidoreductase family protein [Methanocorpusculum labreanum]
>Methanococcus_YdhV CAF30501.1 glyceraldehyde-3-phosphate ferredoxin oxidoreductase [Methanococcus maripaludis S2]
>MethanolineaWP_007313964.1 aldehyde ferredoxin oxidoreductase family protein [Methanolinea tarda]
>Methanomassiliicoccales1 TET89282.1 MAG: hypothetical protein E3J35_10345 [Methanomassiliicoccales archaeon]
>Methanoperedens2 KCZ72477.1 aldehyde:ferredoxin oxidoreductase [Candidatus Methanoperedens nitroreducens]
>Methanosarcina_AAM05121.1 aldehyde ferredoxin oxidoreductase [Methanosarcina acetivorans C2A]
>MethanosarcinaWP_011034557.1 aldehyde ferredoxin oxidoreductase family protein [Methanosarcina mazei]
>MethanosphaerulaWP_012618195.1 aldehyde ferredoxin oxidoreductase family protein [Methanosphaerula palustris]
>MethylibiumWP_011830166.1 aldehyde ferredoxin oxidoreductase family protein [Methylibium petroleiphilum]
>MethylomirabilisCBE69870.1 Tungsten-containing aldehyde ferredoxin oxidoreductase [Candidatus Methylomirabilis oxygeniifera]
>MethylotuvimicrobiumWP_014147264.1 aldehyde ferredoxin oxidoreductase family protein [Methylotuvimicrobium alcaliphilum]
>Methyloversatilis_discipulorum_AOR WP_020164287
>Methyloversatilis_universalis_AOR WP_018229612.1
>MethyloversatilisWP_069037846.1 aldehyde ferredoxin oxidoreductase family protein [Methyloversatilis sp. RAC08]
>MoorellaYP_429035.1 aldehyde ferredoxin oxidoreductase [Moorella thermoacetica ATCC 39073]
>MoorellaYP_429583.1 aldehyde ferredoxin oxidoreductase [Moorella thermoacetica ATCC 39073]
>Mtoga AEX85551.1 aldehyde:ferredoxin oxidoreductase [Marinitoga piezophila KA3]
>NanopusillusAMD29964.1 aldehyde:ferredoxin oxidoreductase [Candidatus Nanopusillus acidilobi]
>NatialbaWP_004214383.1 aldehyde ferredoxin oxidoreductase family protein [Natrialba magadii]
>NatranaerobiusWP_012448479.1 aldehyde ferredoxin oxidoreductase family protein [Natranaerobius thermophilus]
>NatrarchaeobaculumWP_086887942.1 aldehyde ferredoxin oxidoreductase family protein [Natrarchaeobaculum aegyptiacum]
>Natroniella1 WP_248618354.1 aldehyde ferredoxin oxidoreductase family protein [Natroniella sulfidigena]
>NatronobacteriumWP_005579790.1 aldehyde ferredoxin oxidoreductase family protein [Natronobacterium gregoryi]
>NatronococcusWP_015321327.1 aldehyde ferredoxin oxidoreductase family protein [Natronococcus occultus]
>NatronomonasWP_011324185.1 aldehyde ferredoxin oxidoreductase family protein [Natronomonas pharaonis]
>NatronomonasWP_011324209.1 aldehyde ferredoxin oxidoreductase family protein [Natronomonas pharaonis]
>Nezhaarchaeales1 MEM4699718.1 aldehyde ferredoxin oxidoreductase family protein [Candidatus Nezhaarchaeales archaeon]
>Nitrososphaerales1 MEM2883739.1 aldehyde ferredoxin oxidoreductase family protein [Nitrososphaerales archaeon]

>Nitrososphaeria2 NIM44592.1 aldehyde ferredoxin oxidoreductase [Nitrososphaeria archaeon]
>OceanithermusWP_013458790.1 aldehyde ferredoxin oxidoreductase family protein [Oceanithermus profundus]
>Ornatilinea WP_075064124.1 aldehyde ferredoxin oxidoreductase family protein [Ornatilinea apprima]
>Palaeococcus_ferrophilus_2 WP_048150992.1 aldehyde ferredoxin oxidoreductase family protein FOR [Palaeococcus ferrophilus]
>ParamagnetospirillumWP_011385299.1 aldehyde ferredoxin oxidoreductase family protein [Paramagnetospirillum magneticum]
>PelolineaWP_116224811.1 aldehyde ferredoxin oxidoreductase family protein [Pelolinea submarina]
>Pelotomaculum_BAF58675.1 aldehyde:ferredoxin oxidoreductase [Pelotomaculum thermopropionicum SI]
>Pelotomaculum_BAF58690.1 aldehyde:ferredoxin oxidoreductase [Pelotomaculum thermopropionicum SI]
>Pelotomaculum_BAF58791.1 aldehyde:ferredoxin oxidoreductase [Pelotomaculum thermopropionicum SI]
>Pelotomaculum_BAF59046.1 aldehyde:ferredoxin oxidoreductase [Pelotomaculum thermopropionicum SI]
>Pelotomaculum_BAF59102.1 aldehyde:ferredoxin oxidoreductase [Pelotomaculum thermopropionicum SI]
>PelotomaculumBAF60590.1 aldehyde:ferredoxin oxidoreductase [Pelotomaculum thermopropionicum SI]
>PelotomaculumBAF61078.1 aldehyde:ferredoxin oxidoreductase [Pelotomaculum thermopropionicum SI]
>Peptoclostridium_acidaminophilum AHM57984.1 tungsten-containing aldehyde ferredoxin oxidoreductase Aor (plasmid) [Peptoclostridium acidaminophilum DSM 3953]
>PeptoclostridiumWP_025436835.1 aldehyde ferredoxin oxidoreductase family protein [Peptoclostridium acidaminophilum]
>PetrotogaWP_012208230.1 aldehyde ferredoxin oxidoreductase family protein [Petrotoga mobilis]
>Pf2 AAL81327.1 formaldehyde:ferredoxin oxidoreductase [Pyrococcus furiosus DSM 3638]
>PkukuAORWP_068321201.1 aldehyde ferredoxin oxidoreductase family protein [Pyrococcus kukulkanii]
>Planctomycetota1 MFC1524929.1 aldehyde ferredoxin oxidoreductase family protein [Planctomycetota bacterium]
>PromineifilumWP_095043374.1 aldehyde ferredoxin oxidoreductase family protein [Candidatus Promineifilum breve]
>Pyrobaculum_neutrophilum ACB40842.1 Aldehyde ferredoxin oxidoreductase [Pyrobaculum neutrophilum V24Sta]
>PyrobaculumWP_011007373.1 aldehyde ferredoxin oxidoreductase family protein [Pyrobaculum aerophilum]
>PyrobaculumWP_011009372.1 aldehyde ferredoxin oxidoreductase family protein [Pyrobaculum aerophilum]
>Pyrococcus_FOR_1b25 pdb|1B25|A Chain A, PROTEIN (FORMALDEHYDE FERREDOXIN OXIDOREDUCTASE)
>PyrococcusWP_010868080.1 aldehyde ferredoxin oxidoreductase [Pyrococcus abyssi]
>PyrococcusWP_010868317.1 tungsten-containing formaldehyde ferredoxin oxidoreductase [Pyrococcus abyssi]
>PyrococcusWP_011013103.1 MULTISPECIES: aldehyde ferredoxin oxidoreductase family protein [Pyrococcus]
>PyrococcusWP_048146458.1 aldehyde ferredoxin oxidoreductase family protein [Pyrococcus abyssi]
>PyrococcusWP_048146547.1 aldehyde ferredoxin oxidoreductase family protein [Pyrococcus abyssi]
>PyrodictiumWP_055409503.1 aldehyde ferredoxin oxidoreductase family protein [Pyrodictium delaneyi]
>PyrodictiumWP_055410605.1 aldehyde ferredoxin oxidoreductase family protein [Pyrodictium delaneyi]
>Pyrolobus WP_014027339.1 aldehyde ferredoxin oxidoreductase family protein [Pyrolobus fumarii]
>PyrolobusWP_014027123.1 aldehyde ferredoxin oxidoreductase family protein [Pyrolobus fumarii]
>RhodoferaxWP_011465127.1 aldehyde ferredoxin oxidoreductase family protein [Rhodoferax ferrireducens]
>SalinigranumWP_103427036.1 aldehyde ferredoxin oxidoreductase family protein [Salinigranum rubrum]
>Schnuerera WP_005585592.1 aldehyde ferredoxin oxidoreductase family protein [Schnuerera ultunensis]
>SebaldellaWP_012861131.1 aldehyde ferredoxin oxidoreductase family protein [Sebaldella termitidis]
>SedimenticolaWP_046858998.1 aldehyde ferredoxin oxidoreductase family protein [Sedimenticola thiotaurini]
>Serpentinicella WP_132847252.1 aldehyde ferredoxin oxidoreductase family protein [Serpentinicella alkaliphila]
>SlackiaWP_012797611.1 aldehyde ferredoxin oxidoreductase family protein [Slackia heliotrinireducens]
>SlackiaWP_012799570.1 aldehyde ferredoxin oxidoreductase family protein [Slackia heliotrinireducens]
>SoehngeniaWP_128425009.1 aldehyde ferredoxin oxidoreductase family protein [Soehngenia sp. W6]
>StaphylothermusWP_013143562.1 aldehyde ferredoxin oxidoreductase family protein [Staphylothermus hellenicus]
>StaphylothermusWP_052833574.1 aldehyde ferredoxin oxidoreductase family protein [Staphylothermus hellenicus]
>Stherm_ WP_011839485.1 aldehyde ferredoxin oxidoreductase family protein [Staphylothermus marinus]
>SulfuritaleaWP_041099116.1 aldehyde ferredoxin oxidoreductase family protein [Sulfuritalea hydrogenivorans]
>SymbiobacteriumWP_011196177.1 aldehyde ferredoxin oxidoreductase family protein [Symbiobacterium thermophilum]
>SymbiobacteriumWP_011197003.1 aldehyde ferredoxin oxidoreductase family protein [Symbiobacterium thermophilum]
>SymbiobacteriumWP_011197395.1 aldehyde ferredoxin oxidoreductase family protein [Symbiobacterium thermophilum]
>SyntrophobacterWP_011699138.1 aldehyde ferredoxin oxidoreductase family protein [Syntrophobacter fumaroxidans]
>SyntrophobacterWP_041441810.1 aldehyde ferredoxin oxidoreductase [Syntrophobacter fumaroxidans]
>SyntrophomonasWP_011641099.1 aldehyde ferredoxin oxidoreductase family protein [Syntrophomonas wolfei]
>SyntrophomonasWP_011641228.1 aldehyde ferredoxin oxidoreductase family protein [Syntrophomonas wolfei]
>SyntrophothermusWP_013176249.1 aldehyde ferredoxin oxidoreductase family protein [Syntrophothermus lipocalidus]
>T_paralv_2 HIP75290.1 aldehyde ferredoxin oxidoreductase [Thermococcus paralvinellae]
>T_sphaeraWP_013128979.1 aldehyde ferredoxin oxidoreductase family protein [Thermosphaera aggregans]
>T_sphaeraWP_052891605.1 aldehyde ferredoxin oxidoreductase family protein [Thermosphaera aggregans]
>TcAOR WP_088862517.1 aldehyde ferredoxin oxidoreductase family protein [Thermococcus celer]
>Tceler WP_088863869.1 aldehyde ferredoxin oxidoreductase family protein [Thermococcus celer]
>Tf_pendens HHP04352.1 aldehyde ferredoxin oxidoreductase [Thermofilum pendens]
>Thauera_hydrothermalis WP_114648990.1 aldehyde ferredoxin oxidoreductase family protein [Thauera hydrothermalis]
>Theion MBU7013002.1 aldehyde ferredoxin oxidoreductase [Theionarchaea archaeon]
>ThermacetogeniumWP_015051104.1 aldehyde ferredoxin oxidoreductase family protein [Thermacetogenium phaeum]
>ThermacetogeniumWP_015051787.1 aldehyde ferredoxin oxidoreductase family protein [Thermacetogenium phaeum]
>ThermacetogeniumWP_037999095.1 aldehyde ferredoxin oxidoreductase C-terminal domain-containing protein [Thermacetogenium phaeum]
>ThermanaerovibrioYP_003316992.1 Aldehyde ferredoxin oxidoreductase [Thermanaerovibrio acidaminovorans DSM 6589]
>ThermanaerovibrioYP_003317318.1 Aldehyde ferredoxin oxidoreductase [Thermanaerovibrio acidaminovorans DSM 6589]
>Thermincola1 MDT3698119.1 aldehyde ferredoxin oxidoreductase family protein [Thermincola sp.]
>ThermincolaWP_083436764.1 MULTISPECIES: aldehyde ferredoxin oxidoreductase family protein [Thermincola]
>ThermococcusWP_011249027.1 aldehyde ferredoxin oxidoreductase family protein [Thermococcus kodakarensis]
>ThermococcusWP_011250017.1 aldehyde ferredoxin oxidoreductase [Thermococcus kodakarensis]
>ThermodesulfatatorWP_013908697.1 aldehyde ferredoxin oxidoreductase family protein [Thermodesulfatator indicus]
>ThermofilumWP_020961973.1 MULTISPECIES: aldehyde ferredoxin oxidoreductase family protein [Thermofilum]
>ThermofilumWP_020962056.1 MULTISPECIES: aldehyde ferredoxin oxidoreductase family protein [Thermofilum]

>ThermogladiusWP_014737274.1 aldehyde ferredoxin oxidoreductase family protein [Thermogladius calderae]
>Thermoplasmata1 UCH88496.1 MAG: aldehyde ferredoxin oxidoreductase family protein [Thermoplasmata archaeon]
>ThermoplasmaWP_010901222.1 aldehyde ferredoxin oxidoreductase family protein [Thermoplasma acidophilum]
>ThermoplasmaWP_010917497.1 aldehyde ferredoxin oxidoreductase family protein [Thermoplasma volcanium]
>Thermoprotei1 RLF15237.1 MAG: aldehyde ferredoxin oxidoreductase, partial [Thermoprotei archaeon]
>Thermoproteus WP_014127642.1 aldehyde ferredoxin oxidoreductase family protein [Thermoproteus tenax]
>ThermoproteusWP_014127283.1 aldehyde ferredoxin oxidoreductase family protein [Thermoproteus tenax]
>ThermoproteusWP_014127642.1 aldehyde ferredoxin oxidoreductase family protein [Thermoproteus tenax]
>ThermovirgaWP_014163373.1 aldehyde ferredoxin oxidoreductase family protein [Thermovirga lienii]
>ThermovirgaWP_014163584.1 aldehyde ferredoxin oxidoreductase family protein [Thermovirga lienii]
>ThermovirgaWP_014163909.1 aldehyde ferredoxin oxidoreductase family protein [Thermovirga lienii]
>ThermovirgaWP_014163922.1 aldehyde ferredoxin oxidoreductase family protein [Thermovirga lienii]
>ThermovirgaWP_014164024.1 aldehyde ferredoxin oxidoreductase family protein [Thermovirga lienii]
>ThermusYP_143418.1 tungsten-containing aldehyde:ferredoxin oxidoreductase [Thermus thermophilus HB8]
>ThermusYP_143646.1 aldehyde:ferredoxin oxidoreductase [Thermus thermophilus HB8]
>Tlitor2 WP_004066104.1 aldehyde ferredoxin oxidoreductase family protein FOR [Thermococcus litoralis]
>TlitorAOR WP_004066796.1 aldehyde ferredoxin oxidoreductase family protein [Thermococcus litoralis]
>TonAOR WP_012572316.1 aldehyde ferredoxin oxidoreductase family protein [Thermococcus onnurineus]
>TsibirAOR WP_015849284.1 aldehyde ferredoxin oxidoreductase family protein [Thermococcus sibiricus]
>Tsp_aggr HEF88015.1 aldehyde ferredoxin oxidoreductase [Thermosphaera aggregans]
>Vulcanisaeta WP_013335214.1 aldehyde ferredoxin oxidoreductase family protein [Vulcanisaeta distributa]
>VulcanisaetaWP_013335175.1 aldehyde ferredoxin oxidoreductase family protein [Vulcanisaeta distributa]
>VulcanisaetaWP_013335214.1 aldehyde ferredoxin oxidoreductase family protein [Vulcanisaeta distributa]
>VulcanisaetaWP_013335619.1 aldehyde ferredoxin oxidoreductase family protein [Vulcanisaeta distributa]
>VulcanisaetaWP_013337285.1 aldehyde ferredoxin oxidoreductase family protein [Vulcanisaeta distributa]
>WOR4_Pyrococcus_furiosus wp_011013103
>WOR5_Pyrococcus_furiosus WP_014835423
>Ydh_Citrobacter WP_060855300.1 aldehyde ferredoxin oxidoreductase [Citrobacter freundii]
>Ydh_Clostridium WP_040212375.1 aldehyde ferredoxin oxidoreductase [Clostridium polynesiense]
>Ydh_Proteus WP_036932925.1 aldehyde ferredoxin oxidoreductase [Proteus vulgaris]
>YdhV_Ecoli BAE76498.1 predicted oxidoreductase [Escherichia coli str. K-12 substr. W3110]

Acknowledgment

The authors acknowledge the financial support provided by Deutsche Forschungsgemeinschaft He2190/15-1 as well as the National Science Center Poland OPUS 2023/51/B/ST4/01224 grant as well as Polish high-performance computing infrastructure PLGrid (HPC Center: ACK Cyfronet AGH) for providing computer facilities and support within computational grant no. PLG/2023/016888.

References

[1] Chan, M. K., Mukund, S., Kletzin, A., Adams, M. W. W., and Rees, D. C. (1995) Structure of a Hyperthermophilic Tungstopterin Enzyme, Aldehyde Ferredoxin Oxidoreductase, *Science 267*, 1463-1469.

[2] Heider, J., Ma, K., and Adams, M. W. (1995) Purification, characterization, and metabolic function of tungsten-containing aldehyde ferredoxin oxidoreductase from the hyperthermophilic and proteolytic archaeon Thermococcus strain ES-1, *J Bacteriol 177*, 4757-4764.

[3] Ni, Y., Hagedoorn, P.-L., Xu, J.-H., Arends, I. W. C. E., and Hollmann, F. (2014) Pyrococcus furiosus-mediated reduction of conjugated carboxylic acids: Towards using syngas as reductant, *J. Mol. Cat. B: Enzymatic 103*, 52-55.

[4] van den Ban, E. C. D., Willemen, H. M., Wassink, H., Laane, C., and Haaker, H. (1999) Bioreduction of carboxylic acids by Pyrococcus furiosus in batch cultures, *Enzyme Microb Tech 25*, 251-257.

[5] George, G. N., Prince, R. C., Mukund, S., and Adams, M. W. W. (1992) Aldehyde ferredoxin oxidoreductase from the hyperthermophilic archaebacterium Pyrococcus furiosus contains a tungsten oxo-thiolate center, *J. Am. Chem. Soc. 114*, 3521-3523.

[6] Ni, Y., Hagedoorn, P. L., Xu, J. H., Arends, I. W., and Hollmann, F. (2012) A biocatalytic hydrogenation of carboxylic acids, *Chem Commun (Camb) 48*, 12056-12058.

[7] Hagedoorn, P.-L. (2019) Steady-state kinetics of the tungsten containing aldehyde: ferredoxin oxidoreductases from the hyperthermophilic archaeon Pyrococcus furiosus, *J Biotechnol 306*, 142-148.
[8] Lemaire, O. N., Belhamri, M., Schevchenko, A., and Wagner, T. (2024) Carbon monoxide-driven bioethanol production operates via a tungsten-dependent catalyst, *bioRxiv*, 2024.2007.2029.605569.
[9] Hensgens, C. M., Hagen, W. R., and Hansen, T. A. (1995) Purification and characterization of a benzylviologen-linked, tungsten-containing aldehyde oxidoreductase from Desulfovibrio gigas, *J Bacteriol 177*, 6195-6200.
[10] White, H., Strobl, G., Feicht, R., and Simon, H. (1989) Carboxylic acid reductase: a new tungsten enzyme catalyses the reduction of non-activated carboxylic acids to aldehydes, *European Journal of Biochemistry 184*, 89-96.
[11] Kalimuthu, P., Hege, D., Winiarska, A., Gemmecker, Y., Szaleniec, M., Heider, J., and Bernhardt, P. V. (2023) Electrocatalytic Aldehyde Oxidation by a Tungsten Dependent Aldehyde Oxidoreductase from Aromatoleum Aromaticum, *Chemistry – A European Journal 29*, e202203072.
[12] Winiarska, A., Hege, D., Gemmecker, Y., Kryściak-Czerwenka, J., Seubert, A., Heider, J., and Szaleniec, M. (2022) Tungsten Enzyme Using Hydrogen as an Electron Donor to Reduce Carboxylic Acids and NAD+, *Acs Catal 12*, 8707-8717.
[13] Winiarska, A., Ramírez-Amador, F., Hege, D., Gemmecker, Y., Prinz, S., Hochberg, G., Heider, J., Szaleniec, M., and Schuller, J. M. (2023) A bacterial tungsten-containing aldehyde oxidoreductase forms an enzymatic decorated protein nanowire, *Science Advances 9*, eadg6689.
[14] Schmitt, G., Arndt, F., Kahnt, J., and Heider, J. (2017) Adaptations to a Loss-of-Function Mutation in the Betaproteobacterium *Aromatoleum aromaticum*: Recruitment of Alternative Enzymes for Anaerobic Phenylalanine Degradation, *J Bacteriol 199*, e00383-00317.
[15] Debnar-Daumler, C., Seubert, A., Schmitt, G., and Heider, J. (2014) Simultaneous Involvement of a Tungsten-Containing Aldehyde: Ferredoxin Oxidoreductase and a Phenylacetaldehyde Dehydrogenase in Anaerobic Phenylalanine Metabolism, *J. Bacteriol. 196*, 483-492.
[16] Luo, S., Adam, D., Giaveri, S., Barthel, S., Cestellos-Blanco, S., Hege, D., Paczia, N., Castañeda-Losada, L., Klose, M., Arndt, F., Heider, J., and Erb, T. J. (2023) ATP production from electricity with a new-to-nature electrobiological module, *Joule 7*, 1745-1758.
[17] Arndt, F., Schmitt, G., Winiarska, A., Saft, M., Seubert, A., Kahnt, J., and Heider, J. (2019) Characterization of an Aldehyde Oxidoreductase From the Mesophilic Bacterium Aromatoleum aromaticum EbN1, a Member of a New Subfamily of Tungsten-Containing Enzymes, *Frontiers in Microbiology 10*, 71.
[18] Putumbaka, S., Schut, G. J., Thorgersen, M. P., Poole, F. L., Shao, N., Rodionov, D. A., and Adams, M. W. W. (2025) Tungsten is utilized for lactate consumption and SCFA production by a dominant human gut microbe Eubacterium limosum, *Proc Natl Acad Sci U S A 122*, e2411809121.
[19] Schut, G. J., Thorgersen, M. P., Poole, F. L., 2nd, Haja, D. K., Putumbaka, S., and Adams, M. W. W. (2021) Tungsten enzymes play a role in detoxifying food and antimicrobial aldehydes in the human gut microbiome, *Proc Natl Acad Sci U S A 118*.
[20] Thorgersen, M. P., Schut, G. J., Poole, F. L., 2nd, Haja, D. K., Putumbaka, S., Mycroft, H. I., de Vries, W. J., and Adams, M. W. W. (2022) Obligately aerobic human gut microbe expresses an oxygen resistant tungsten-containing oxidoreductase for detoxifying gut aldehydes, *Front Microbiol 13*, 965625.
[21] Reher, M., Gebhard, S., and Schonheit, P. (2007) Glyceraldehyde-3-phosphate ferredoxin oxidoreductase (GAPOR) and nonphosphorylating glyceraldehyde-3-phosphate dehydrogenase (GAPN), key enzymes of the respective modified Embden-Meyerhof pathways in the hyperthermophilic crenarchaeota Pyrobaculum aerophilum and Aeropyrum pernix, *FEMS Microbiol Lett 273*, 196-205.

[22] Mukund, S., and Adams, M. W. W. (1995) Glyceraldehyde-3-phosphate Ferredoxin Oxidoreductase, a Novel Tungsten-containing Enzyme with a Potential Glycolytic Role in the Hyperthermophilic Archaeon Pyrococcus furiosus*, *Journal of Biological Chemistry 270*, 8389-8392.
[23] Park, M. O., Mizutani, T., and Jones, P. R. (2007) Glyceraldehyde-3-phosphate ferredoxin oxidoreductase from Methanococcus maripaludis, *J Bacteriol 189*, 7281-7289.
[24] Scott, I. M., Rubinstein, G. M., Poole, F. L., 2nd, Lipscomb, G. L., Schut, G. J., Williams-Rhaesa, A. M., Stevenson, D. M., Amador-Noguez, D., Kelly, R. M., and Adams, M. W. W. (2019) The thermophilic biomass-degrading bacterium Caldicellulosiruptor bescii utilizes two enzymes to oxidize glyceraldehyde 3-phosphate during glycolysis, *J Biol Chem 294*, 9995-10005.
[25] Hagedoorn, P. L., Freije, J. R., and Hagen, W. R. (1999) Pyrococcus furiosus glyceraldehyde 3-phosphate oxidoreductase has comparable W(6+/5+) and W(5+/4+) reduction potentials and unusual [4Fe-4S] EPR properties, *FEBS Lett 462*, 66-70.
[26] Scott, I. M., Rubinstein, G. M., Lipscomb, G. L., Basen, M., Schut, G. J., Rhaesa, A. M., Lancaster, W. A., Poole, F. L., Kelly, R. M., and Adams, M. W. W. (2015) A New Class of Tungsten-Containing Oxidoreductase in Caldicellulosiruptor, a Genus of Plant Biomass-Degrading Thermophilic Bacteria, *Applied and Environmental Microbiology 81*, 7339-7347.
[27] Hu, Y. L., Faham, S., Roy, R., Adams, M. W. W., and Rees, D. C. (1999) Formaldehyde ferredoxin oxidoreductase from Pyrococcus furiosus: The 1.85 angstrom resolution crystal structure and its mechanistic implications, *J Mol Biol 286*, 899-914.
[28] Bol, E., Broers, N. J., and Hagen, W. R. (2008) A steady-state and pre-steady-state kinetics study of the tungstoenzyme formaldehyde ferredoxin oxidoreductase from Pyrococcus furiosus, *J Biol Inorg Chem 13*, 75-84.
[29] Mathew, L. G., Haja, D. K., Pritchett, C., McCormick, W., Zeineddine, R., Fontenot, L. S., Rivera, M. E., Glushka, J., Adams, M. W. W., and Lanzilotta, W. N. (2022) An unprecedented function for a tungsten-containing oxidoreductase, *J Biol Inorg Chem 27*, 747-758.
[30] Bevers, L. E., Bol, E., Hagedoorn, P. L., and Hagen, W. R. (2005) WOR5, a novel tungsten-containing aldehyde oxidoreductase from Pyrococcus furiosus with a broad substrate Specificity, *J Bacteriol 187*, 7056-7061.
[31] Seelmann, C. S., Huwiler, S. G., Culka, M., Strampraad, M. J. F., Biskup, T., Weber, S., Ullmann, G. M., Schünemann, V., Hagedoorn, P.-L., Pierik, A. J., and Boll, M. (2023) Enzymatic Birch Reduction via Hydrogen Atom Transfer at an Aqua-Tungsten-bis-Metallopterin Cofactor, *Acs Catal 13*, 8631-8641.
[32] Weinert, T., Huwiler, S. G., Kung, J. W., Weidenweber, S., Hellwig, P., Stark, H. J., Biskup, T., Weber, S., Cotelesage, J. J., George, G. N., Ermler, U., and Boll, M. (2015) Structural basis of enzymatic benzene ring reduction, *Nat Chem Biol 11*, 586-591.
[33] Kung, J. W., Baumann, S., von Bergen, M., Muller, M., Hagedoorn, P. L., Hagen, W. R., and Boll, M. (2010) Reversible Biological Birch Reduction at an Extremely Low Redox Potential, *Journal of the American Chemical Society 132*, 9850-9856.
[34] Loffler, C., Kuntze, K., Vazquez, J. R., Rugor, A., Kung, J. W., Bottcher, A., and Boll, M. (2011) Occurrence, genes and expression of the W/Se-containing class II benzoyl-coenzyme A reductases in anaerobic bacteria, *Environ Microbiol 13*, 696-709.
[35] Culka, M., Huwiler, S. G., Boll, M., and Ullmann, G. M. (2017) Breaking Benzene Aromaticity-Computational Insights into the Mechanism of the Tungsten-Containing Benzoyl-CoA Reductase, *J Am Chem Soc 139*, 14488-14500.
[36] Kroneck, P. M. (2016) Acetylene hydratase: a non-redox enzyme with tungsten and iron-sulfur centers at the active site, *J Biol Inorg Chem 21*, 29-38.
[37] Meckenstock, R. U., Krieger, R., Ensign, S., Kroneck, P. M. H., and Schink, B. (1999) Acetylene hydratase of Pelobacter acetylenicus - Molecular and spectroscopic

properties of the tungsten iron-sulfur enzyme, *European Journal of Biochemistry 264*, 176-182.

[38] Seiffert, G. B., Ullmann, G. M., Messerschmidt, A., Schink, B., Kroneck, P. M. H., and Einsle, O. (2007) Structure of the non-redox-active tungsten/ 4Fe : 4S enzyme acetylene hydratase, *Proc Natl Acad Sci U S A 104*, 3073-3077.

[39] tenBrink, F., Schink, B., and Kroneck, P. M. H. (2011) Exploring the Active Site of the Tungsten, Iron-Sulfur Enzyme Acetylene Hydratase, *J Bacteriol 193*, 1229-1236.

[40] Sievers, F., Wilm, A., Dineen, D., Gibson, T. J., Karplus, K., Li, W., Lopez, R., McWilliam, H., Remmert, M., Söding, J., Thompson, J. D., and Higgins, D. G. (2011) Fast, scalable generation of high-quality protein multiple sequence alignments using Clustal Omega, *Molecular Systems Biology 7*, 539.

[41] Letunic, I., and Bork, P. (2024) Interactive Tree of Life (iTOL) v6: recent updates to the phylogenetic tree display and annotation tool, *Nucleic Acids Res 52*, W78-W82.
